# Clinicopathological and Demographical Characteristics of Non-Small Cell Lung Cancer Patients with ALK Rearrangements: A Systematic Review and Meta-Analysis

**DOI:** 10.1371/journal.pone.0100866

**Published:** 2014-06-24

**Authors:** Liang Fan, Yun Feng, Huanying Wan, Guochao Shi, Wenquan Niu

**Affiliations:** 1 Department of Respiratory Medicine, Ruijin Hospital, School of Medicine, Shanghai Jiao Tong University, Shanghai, China; 2 State Key Laboratory of Medical Genomics, Ruijin Hospital, School of Medicine, Shanghai Jiao Tong University, Shanghai, China; Ottawa Hospital Research Institute, Canada

## Abstract

**Objective:**

This meta-analysis aimed to comprehensively examine the relationship between the clinicopathological and demographical characteristics and ALK rearrangements in patients with non-small cell lung cancer (NSCLC).

**Methods and Main Findings:**

In total, 62 qualified articles including 1178 ALK rearranged cases from 20541 NSCLC patients were analyzed, and the data were extracted independently by two investigators. NSCLC patients with ALK rearrangements tended to be younger than those without (mean difference: −7.16 years; 95% confidence interval (95% CI): −9.35 to −4.96; P<0.00001), even across subgroups by race. Compared with female NSCLC patients, the odds ratio (OR) of carrying ALK rearrangements was reduced by 28% (95% CI: 0.58–0.90; P = 0.004) in males, and this reduction was potentiated in Asians, yet in opposite direction in Caucasians. Likewise, smokers were less likely to have ALK rearrangements than never-smokers (OR = 0.33; 95% CI: 0.25–0.44; P<0.00001), even in race-stratified subgroups. Moreover, compared with NSCLC patients with tumor stage IV, ALK rearrangements were underrepresented in those with tumor stage I–III (OR = 0.58; 95% CI: 0.44–0.78; P = 0.0002). Patients with lung adenocarcinomas had a significantly higher rate of ALK rearrangements (7.2%) than patients with non-adenocarcinoma (2.0%) (OR = 2.25; 95% CI: 1.54–3.27; P<0.0001).

**Conclusion:**

Our findings demonstrate that ALK rearrangements tended to be present in NSCLC patients with no smoking habit, younger age and tumor stage IV. Moreover, race, age, gender, smoking status, tumor stage and histology might be potential sources of heterogeneity.

## Introduction

Lung cancer is the leading cause of cancer deaths worldwide. Most of lung cancer patients are diagnosed at an advanced stage with extremely poor prognoses. Non-small cell lung cancer (NSCLC) accounts for approximately 80% of all lung cancers. With the advancements of medical science, much hope has been laid on pharmacogenomics as a novel approach to circumvent problems in individualized medical therapy for cancer. For example, NSCLC patients with activating mutations in epidermal growth factor receptor (EGFR) gene had a good response to its tyrosine kinase inhibitors [1]. In 2007, Soda and colleagues first identified a tyrosine kinase as a promising therapeutic target and diagnostic molecular marker for NSCLC, and this kinase accelerates the formation of a fusion gene comprising the portions of echinoderm microtubule-associated protein-like 4 (EML4) and the anaplastic lymphoma kinase (ALK) in NSCLC cells [2]. This formation is biologically important as EML4 activates ALK kinase via mediating ligand-independent oligomerization of ALK [3], [4], and the activated ALK is responsible for the growth and survival of lung cancer cell lines, the process being highly sensitive to ALK kinase inhibitors [5]. Several clinical data have reported that administration of crizotinib, an inhibitor of ALK tyrosine kinases, was beneficial to lung cancer patients with ALK rearrangements [6], [7]. Epidemiologic studies showed that ALK rearrangements tended to occur in younger patients, never or light smokers and patients with adenocarcinoma rather than squamous cell or large cell carcinoma [8], [9]. In addition, there was evidence for a mutually exclusive condition between ALK rearrangements and EGFR or KRAS mutations [10]. It is estimated that the incidence of ALK rearrangements in unselected NSCLC populations is 2%–7% [9], [11], [12], indicating that only a small proportion of NSCLC patients will benefit from ALK kinase inhibitors, and the accurate and timely identification of these patients will have important therapeutic implications. Therefore, understanding the clinicopathological characteristics of ALK rearrangements will be a major requirement for optimal management of NSCLC patients. However, a comprehensive evaluation of these characteristics so far is lacking in medical literature. Given the accumulating data, there is an urgent need to synthesize available articles by means of a meta-analysis to comprehensively examine the relationship between the clinicopathological and demographical characteristics and ALK rearrangements in NSCLC patients.

## Materials and Methods

We carried out this meta-analysis of cross-sectional studies in accordance with the guidelines set forth by the Preferred Reporting Items for Systematic Reviews and Meta-analyses (PRISMA) statement (see Checklist S1) [13].

### Search strategy for identification of studies

We searched PubMed and EMBASE (Excerpta Medica database) for articles published before December 10, 2013. Subject terms included anaplastic lymphoma kinase or ALK and lung cancer. Search results were expressed in Boolean expression: ((anaplastic lymphoma kinase) OR ALK) AND (lung cancer)) AND English [Language].

### Study selection

Two investigators (L.F. and Y.F.) independently obtained the full texts of potentially eligible articles based on their titles and abstracts. To avoid the double counting of the patients recruited in more than one publication, the authors were contacted for inquiry when necessary. Where more than one publication of a study population existed, we extracted information from the most recent or most complete publication.

### Inclusion/exclusion criteria

Articles were included if they met the following criteria: (1) NSCLC was diagnosed based on either histological or cytological results; (2) ALK rearrangements were determined in ALK-status unknown NSCLC patients by using FISH or IHC or PCR method; (3) one or more clinicopathological or demographical characteristics including age, gender, smoking habit, tumor stage, histology and EGFR/KRAS mutation status were provided between ALK-rearranged and ALK negative NSCLC patients. Articles were excluded if they lacked valid data for comparisons between patients with and without ALK rearrangements across clinicopathological or demographical characteristics, or if they were conference abstracts/proceedings, case reports/series, editorials, narrative reviews and the non-English articles.

### Data extraction

Two authors (L.F. and Y.F.) of this study independently extracted the following information: the first author's name, year of publication, sample size, the method to detect ALK rearrangements, tumor histology and stage, age, gender, race and smoking status if available. The discrepancies were resolved by the discussion and review of original articles, and a consensus was reached finally.

### Statistical analysis

The meta-analysis was conducted using the open-source Review Manager (RevMan) Software (version 5.2.4, available at the website http://ims.cochrane.org/revman/download). Irrespective of the presence of heterogeneity between studies, the random-effects model was employed to combine individual effect-size estimates. The relationship between clinicopathological or demographical profiles including gender, smoking status, tumor stage, histology and ALK rearrangements were assessed by inverse variance (IV) method, and effect estimates were expressed as odds ratio (OR) [14] or weighted mean difference (WMD) and 95% confidence interval (95% CI). Age difference was estimated with inverse variance method and contrasts were expressed in the form of mean difference and 95% CI. For those articles with only median age and age range, we estimated mean age and standard deviation using the methods described by Hozo [15].

Heterogeneity was examined using the inconsistency index (*I* ^2^) statistic, which ranges from 0% to 100% and is defined as the percentage of the observed between-trial variability that is due to heterogeneity rather than chance. Generally, *I* ^2^>50% was used as a threshold indicating significant heterogeneity. Meta-regression analysis was carried out to evaluate the extent to which different study-level variables including clinicopathological or demographical characteristics as mentioned above explained the heterogeneity of pooled effect estimates.

Publication bias was assessed by the fail-safe number (N_fs_). If the N_fs_ was smaller than the number of observed studies for the same comparison, this was interpreted as meaning that mete-analysis result might have a significant publication bias as previously described [16]. The N_fs_ was calculated as 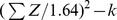, where k is equal to the number of articles involved in calculation.

## Results

### Qualified articles

**Figure 1:**
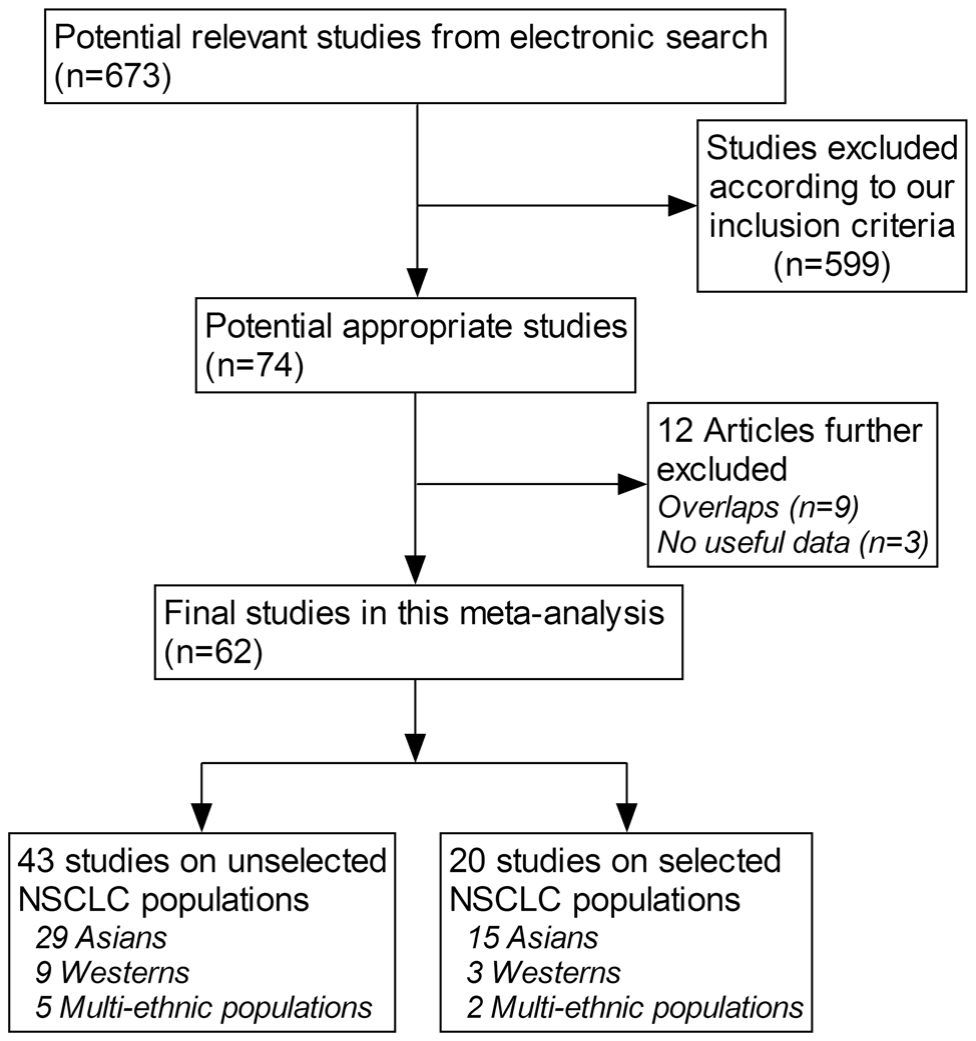
Flow diagram of search strategy and study selection.

Based on the search strategy, a total of 20541 NSCLC patients were analyzed from 62 qualified articles [4],[8],[11],[12],[17]–[74], and of them 1178 patients (5.7%) had ALK rearrangements. 43 of 62 articles were conducted in Asians (19 in Chinese, 13 in Japanese, 10 in Koreans and 1 in Indians), 12 articles in Caucasians (7 in Americans and 5 in Europeans) and 7 articles in multi-ethnic populations. 43 articles involved unselected populations [4], [8], [11], [12], [17]–[55], and 20 articles enrolled specific groups of NSCLC patients according to either clinicopathological characteristics or genetic makeup [46], [56]–[74], with one article [46] provided data from both unselected and selected NSCLC groups. A flow diagram schematizing the process of excluding articles with specific reasons is presented in Figure 1.

### Characteristics

The characteristics of all qualified articles are summarized in Table 1 and Table S1. The incidence of ALK rearrangements ranged from 0.99% to 15.00% in unselected NCSLC groups based on 43 articles (4.77% in 29 East Asian groups; 3.91% in 9 Caucasian groups; 5.15% in 5 multi-ethnic groups). For selected NSCLC groups according to gender, smoking status, tumor stage, or EGFR mutation, the incidence of ALK rearrangements ranged from 4.30% to 34.78% based on 20 articles (15 in East Asians; 3 in Caucasians; 2 in multi-ethnic groups).

**Table 1:** Clinicopathological and demographical characteristics of NSCLC patients with ALK rearrangements.

Comparisons	Subgroups		Articles	Characteristics	Effect estimate (95% CI)	p value	df(p)	*I* ^2^
Gender	NSCLC	Western	9	males (59/559, 10.6%) vs. females (54/536, 10.1%)	OR: 1.12 (0.55, 2.28)	0.76	8(p = 0.05)	49%
		Asian	36	males (282/6492, 4.3%) vs. females (381/5070, 7.5%)	OR: 0.59 (0.49, 0.73)	<0.00001	36(p = 0.09)	24%
		Mixed	5	males (56/638, 8.8%) vs. females (72/1041, 6.9%)	OR: 1.51 (0.86, 2.64)	0.15	4(p = 0.10)	49%
		Total	50	males (397/7689, 5.2%) vs. females (507/6647, 7.6%)	OR: 0.72 (0.58, 0.90)	0.004	50(p<0.0001)	51%
	Ad only	Western	4	males (25/207, 12.1%) vs. females (20/281, 7.2%)	OR: 1.70 (0.64, 4.50)	0.29	3(p = 0.18)	39%
		Asian	13	males (136/2130, 6.4%) vs. females (160/2196, 7.3%)	OR: 0.83 (0.65, 1.06)	0.13	12(0.97)	0%
		Mixed	2	males (30/305, 9.8%) vs. females (48/670, 7.2%)	OR: 1.44 (0.66, 3.13)	0.36	1(p = 0.13)	56%
		Total	19	males (191/2642, 7.2%) vs. females (228/3147, 7.2%)	OR: 1.00 (0.79, 1.25)	0.97	18(p = 0.29)	13%
Smoking status	NSCLC	Western	6	smoker (27/668, 3.6%) vs. non-smoker (57/243, 23.5%)	OR: 0.16 (0.08, 0.32)	<0.00001	5(p = 0.25)	24%
		Asian	26	smoker (142/4363, 3.3%) vs. non-smoker (309/3724, 8.3%)	OR: 0.42 (0.31, 0.58)	<0.00001	25(p = 0.01)	43%
		Mixed	4	smoker (25/829, 3.0%) vs. non-smoker (67/492, 13.6%)	OR: 0.19 (0.12, 0.32)	<0.00001	3(0.99)	0%
		Total	36	smoker (194/5860, 3.3%) vs. non-smoker (433/4459, 9.7%)	OR: 0.33 (0.25, 0.44)	<0.00001	35(p = 0.0007)	49%
	Ad only	Western	2	smoker (6/296, 2.0%) vs. non-smoker (19/97, 19.6%)	OR: 0.06 (0.01, 0.52)	<0.00001	1(p = 0.15)	52%
		Asian	11	smoker (63/1448, 4.4%) vs. non-smoker (160/1922, 8.3%)	OR: 0.54 (0.39, 0.75)	= 0.0002	10(p = 0.41)	3%
		Mixed	1	smoker (9/382, 2.4%) vs. non-smoker (35/293, 11.9%)	OR: 0.18 (0.08, 0.38)	<0.00001	NA	NA
		Total	14	smoker (78/2126, 3.7%) vs. non-smoker (214/2312, 9.3%)	OR: 0.36 (0.23, 0.57)	<0.0001	13(p = 0.008)	54%
Tumor stage	NSCLC	Western	3	stage I–III (13/453, 2.9%) vs. stage IV (18/131, 13.7%)	OR: 0.21 (0.04, 0.21)	0.05	2(p = 0.13)	51%
		Asian	19	stage I–III (248/5652, 4.4%) vs. stage IV (101/929, 10.9%)	OR: 0.70 (0.51, 0.98)	0.04	18(p = 0.95)	0%
		Mixed	4	stage I–III (41/639, 6.4%) vs. stage IV (43/345, 12.5%)	OR: 0.57 (0.30, 1.11)	0.10	3(p = 0.65)	0%
		Total	26	stage I–III (302/6744, 4.5%) vs. stage IV (162/1405, 11.5%)	OR: 0.58 (0.44, 0.78)	0.0002	25(p = 0.43)	2%
	Ad only	Western	1	stage I–III (4/265, 1.5%) vs. stage IV (16/93, 17.2%)	OR: 0.07 (0.02, 0.23)	<0.00001	NA	NA
		Asian	7	stage I–III (87/1727, 5.0%) vs. stage IV (22/222, 9.9%)	OR: 0.73 (0.40, 1.33)	0.31	6(p = 0.83)	0%
		Mixed	1	stage I–III (28/253, 11.1%) vs. stage IV (6/47, 12.8%)	OR: 0.85 (0.33, 2.18)	0.74	NA	NA
		Total	9	stage I–III (119/2245, 5.3%) vs. stage IV (44/362, 12.1%)	OR: 0.53 (0.25, 1.09)	0.09	8(p = 0.03)	52%
Histology	Ad vs. non-Ad	Western	7	Ad (100/1654, 6.0%) vs. non-Ad (23/677, 3.4%)	OR: 1.11 (0.59, 2.07)	0.75	6(p = 0.30)	18%
		Asian	23	Ad (302/3997, 7.6%) vs. non-Ad (28/1898, 1.5%)	OR: 2.92 (1.88, 4.53)	<0.00001	22(p = 0.27)	14%
		Mixed	3	Ad (47/549, 8.6%) vs. non-Ad (3/149, 2.0%)	OR: 2.44 (0.79, 7.48)	0.12	2(p = 0.63)	0%
		Total	33	Ad (449/6200, 7.2%) vs. non-Ad (54/2724, 2.0%)	OR: 2.25 (1.54, 3.27)	<0.0001	32(p = 0.09)	26%
	Ad vs. SCC	Western	5	Ad (24/443, 5.4%) vs. Scc (5/241, 2.1%)	OR: 1.33 (0.48, 3.68)	0.58	4(p = 0.60)	0%
		Asian	19	Ad (227/3594, 6.3%) vs. Scc (11/1415, 0.8%)	OR: 3.64 (2.17, 6.09)	<0.00001	18(p = 0.67)	0%
		Mixed	3	Ad (47/549, 8.6%) vs. Scc (1/98, 1.0%)	OR: 1.46 (0.33, 6.54)	0.62	2(p = 0.37)	0%
		Total	27	Ad (298/4586, 6.5%) vs. Scc (17/1754, 1.0%)	OR: 2.79 (1.80, 4.33)	<0.00001	26(p = 0.57)	0%
Age	NSCLC	Western	8	mean age: ALK(+) (54.5, 104) vs. ALK(−) (64.2, 872)	WMD: −6.18 (−15.13, 2.77)	0.18	7(p<0.00001)	96%
		Asian	28	mean age: ALK(+) (54.6, 519) vs. ALK(−) (62.9, 8647)	WMD: −6.99 (−8.91, −5.08)	<0.00001	27(p<0.00001)	74%
		Mixed	4	mean age: ALK(+) (57.3, 120) vs. ALK(−) (66.0, 1248)	WMD: −9.57 (−13.28, −5.86)	<0.00001	3(p = 0.06)	59%
		Total	40	mean age: ALK(+) (55.1, 743) vs. ALK(−) (63.4, 10767)	WMD: −7.16 (−9.35, −4.96)	<0.00001	39(p<0.00001)	87%
	Ad only	Western	4	mean age: ALK(+) (57.0, 45) vs. ALK(−) (66.1, 444)	WMD: −8.62 (−19.27, 2.01)	0.11	3(p<0.00001)	92%
		Asian	13	mean age: ALK(+) (57.9, 242) vs. ALK(−) (62.9, 3826)	WMD: −5.40(−7.35, −3.46)	<0.00001	12(p = 0.01)	52%
		Mixed	3	mean age: ALK(+) (58.2, 101) vs. ALK(−) (66.2, 1126)	WMD: −8.98 (−13.52, −4.44)	0.0001	2(p = 0.02)	74%
		Total	20	mean age: ALK(+) (57.9, 388) vs. ALK(−) (63.8, 5396)	WMD: −6.81 (−9.18, −4.45)	<0.00001	19(p<0.00001)	80%
	ALK vs. EGFR	Western	2	mean age: ALK(+) (51.9, 48) vs. EGFR mutated (61.5, 52)	WMD: −8.26 (−15.94, −0.57)	0.04	1(p = 0.17)	47%
		Asian	9	mean age: ALK(+) (54.1, 110) vs. EGFR mutated (59.7, 891)	WMD: −5.89 (−9.62, −2.16)	0.002	8(p = 0.0005)	72%
		Mixed	4	mean age: ALK(+) (56.5, 161) vs. EGFR mutated (64.4, 582)	WMD: −8.17 (−11.17, −5.17)	<0.00001	3(p = 0.13)	46%
		Total	15	mean age: ALK(+) (55.0, 319) vs. EGFR mutated (61.5, 1525)	WMD: −6.95 (−9.32, −4.59)	<0.00001	14(p = 0.0003)	64%
	ALK vs. KRAS	Western	1	mean age: ALK(+) (51, 41) vs. KRAS mutated (59.5, 49)	WMD: −8.50 (−14.09, −2.91)	0.003	NA	NA
		Asian	6	mean age: ALK(+) (56.3, 67) vs. KRAS mutated (60.6, 66)	WMD: −4.26 (−7.70, −0.82)	0.02	5(p = 0.46)	0%
		Mixed	3	mean age: ALK(+) (57.1, 142) vs. KRAS mutated (65.8, 707)	WMD: −8.54 (−11.53, −5.56)	<0.00001	2(p = 0.16)	46%
		Total	10	mean age: ALK(+) (55.9, 250) vs. KRAS mutated (65.0, 822)	WMD: −6.94 (−9.27, −4.60)	<0.00001	9(p = 0.13)	35%

*Abbreviations:* NSCLC, non-small-cell lung cancer; Ad, adenocarcinoma; SCC, squamous cell carcinoma; OR, odds ratio; WMD, weighted mean difference; CI, confidence interval.

### ALK rearrangements and age

**Figure 2:**
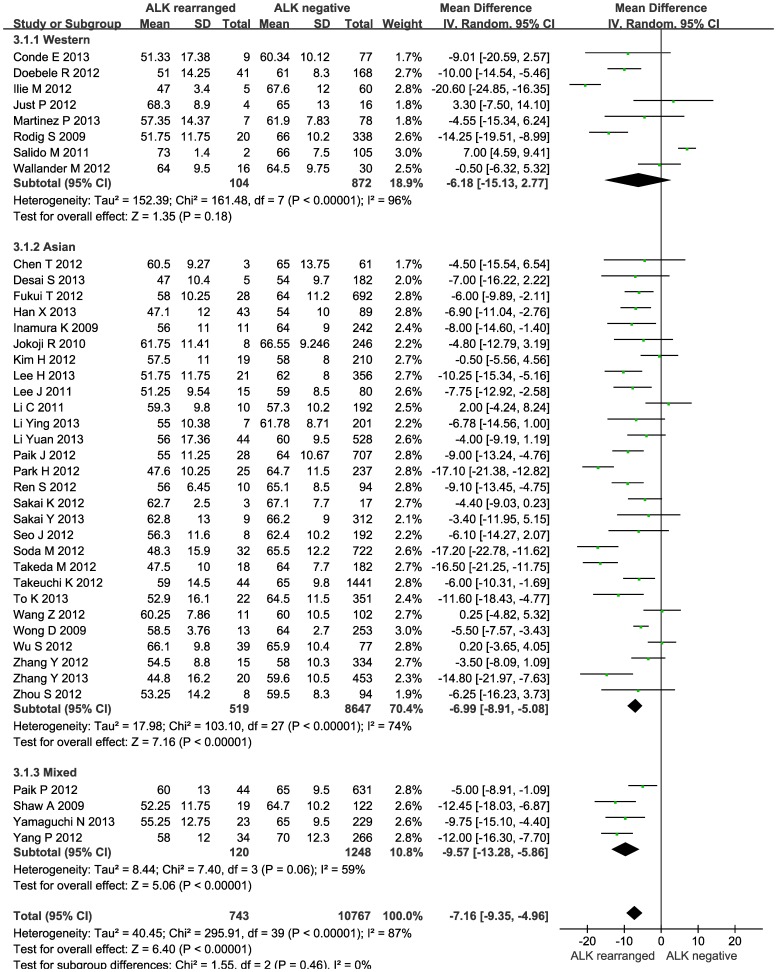
Forest plots of the mean difference of age between NSCLC patients with and without ALK rearrangements by race.

NSCLC patients with ALK rearrangements tended to be younger than those without (WMD: −7.16 years; 95% CI: −9.35 to −4.96; P<0.00001), and this tendency was persisted in Asians (WMD: −6.99; 95% CI: −8.91 to −5.08; P<0.00001) and Caucasians (WMD: −6.18, 95% CI: −15.13 to 2.77; P = 0.18), albeit strong evidence of heterogeneity (Figure 2). Analyzing patients with only lung adenocarcinomas observed similar results with significant heterogeneity for age in Asians (WMD: −5.40; 95% CI: −7.35 to −3.46; P<0.00001) and Caucasians (WMD: −8.62; 95% CI: −19.27 to 2.01, P = 0.11) (Figure S1). As indicated by the N_fs_, there was no observable publication bias.

### ALK rearrangements and gender

**Figure 3:**
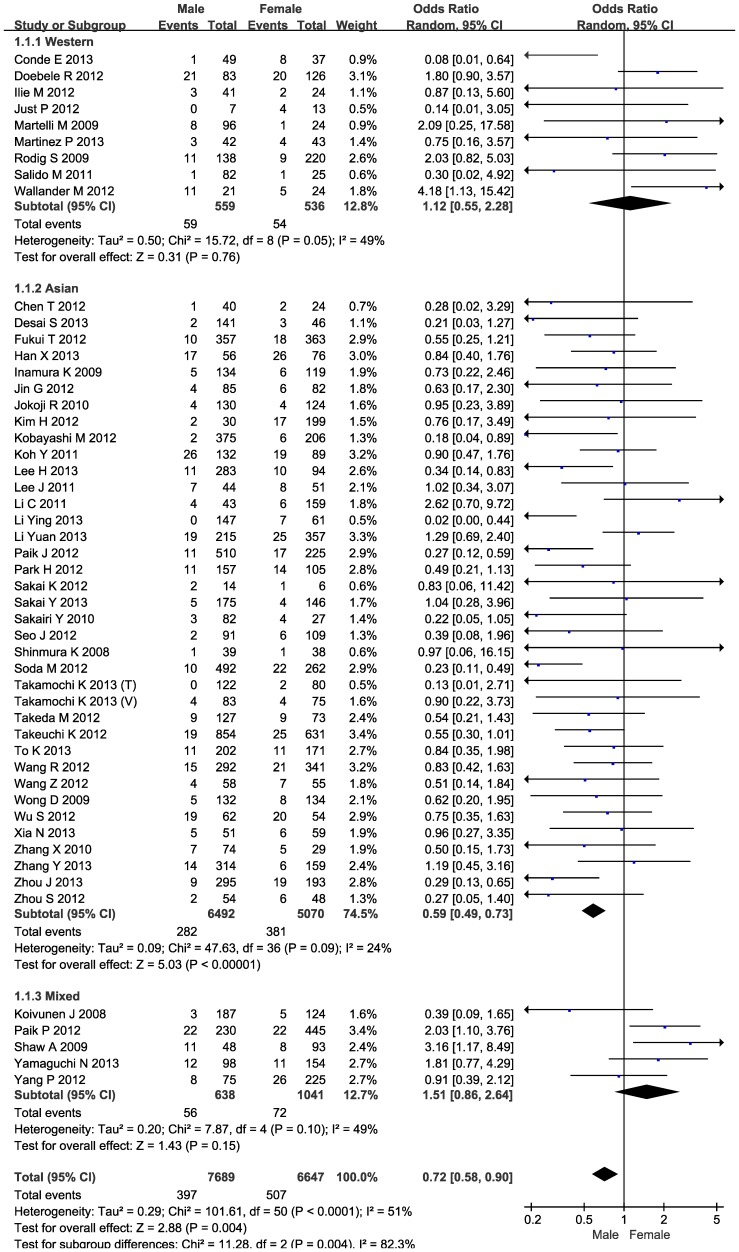
Forest plots of gender difference between NSCLC patients with and without ALK rearrangements by race.

The frequencies of ALK rearrangements ranged respectively from 0% to 30.65% and from 2.63% to 37.04% in male (397/7689) and female (507/6627) patients with NSCLC based on 50 articles. Compared with female patients with NSCLC, the odds of carrying ALK rearrangements was reduced by 28% (95% CI: 0.58–0.90; P = 0.004) in males (*I* ^2^ = 51%) (Figure 3). In subgroup analysis by race, further significant reduction in odds was observed in male patients of Asian descent (OR = 0.59; 95% CI: 0.49–0.73; P<0.00001), but there was an elevated yet nonsignificant risk in male patients of Caucasian descent (OR = 1.12; 95% CI: 0.55–2.28; P = 0.76) with no heterogeneity or publication bias (Figure 3). After limiting articles to patients with lung adenocarcinomas (n = 19), risk estimates were similar in direction for male patients of Asian descent (OR = 0.83; 95% CI: 0.65–1.06; P = 0.13) and Caucasian descent (OR = 1.70; 95% CI: 0.64–4.50; P = 0.29), and there was no observable heterogeneity (Figure S2); however, the N_fs_ values were less than the number of articles in each comparison, indicating the presence of publication bias.

### ALK rearrangements and smoking status

The frequencies of ALK rearrangements in NSCLC patients ranged from 0% to 19.44% and from 0% to 41.67% among smokers and never-smokers, respectively. Smokers were less likely to have ALK rearrangements than never-smokers (OR = 0.33; 95% CI: 0.25–0.44; P<0.00001), even in subgroups by race, such as in Asians (OR = 0.42; 95% CI: 0.31–0.58; P<0.00001) and Caucasians (OR = 0.16; 95% CI: 0.08–0.32; P<0.00001) (Figure S3), and there was no heterogeneity and low probabilities of publication bias as reflected by the N_fs_. Similarly in patients with only lung adenocarcinomas (14 qualified articles), smokers still had reduced risk compared with never-smokers (OR = 0.36; 95% CI: 0.23–0.57; P<0.00001; *I* ^2^ = 54%), even in East Asian patients (OR = 0.54; 95% CI: 0.38–0.75; P<0.00001; *I* ^2^ = 0%) (Figure S4), and still heterogeneity and publication bias were unlikely for these comparisons.

### ALK rearrangements and tumor stage

ALK rearrangements were detected in 3.85%, 4.31%, 5.72% and 12.39% of NSCLC patients with tumor stage of I (30 articles: 199/5168), II (25 articles: 50/1161), III (27 articles: 119/2080) and IV (28 articles: 214/1727), respectively (Table S2). Compared with NSCLC patients with stage IV, ALK rearrangements were underrepresented in NSCLC patients with stage I–III (OR = 0.58; 95% CI: 0.44–0.78; P = 0.0002), without heterogeneity (*I* ^2^ = 2%). The ORs were 0.7 (95% CI: 0.51–0.98; P = 0.04) and 0.21 (95% CI: 0.04–0.21; P = 0.05) in East Asians (*I* ^2^ = 0%) and Caucasians (*I* ^2^ = 51%), respectively (Figure S5), and this finding was unlikely explained by publication bias. When only lung adenocarcinomas was concerned, NSCLC patients with stage I–III had lower yet nonsignificant incidence of ALK rearrangements than those with stage IV (OR = 0.53; 95% CI: 0.25–1.09; P = 0.09), with moderate heterogeneity (*I* ^2^ = 52%) and evident publication bias (N_fs_ = −1.14) (Figure S6).

### ALK rearrangements and histology

ALK rearrangements were observed in 6.92% (55 articles: 918/13275), 0.92% (26 articles: 16/1746), 11.54% (13 articles: 9/78) and 5.97% (13 articles: 4/67) patients with lung adenocarcinomas, squamous carcinomas, adenosquamous carcinomas and large cell carcinomas, respectively (Table S3). Patients with lung adenocarcinomas had a significantly higher rate of ALK rearrangements (449/6200; 7.2%) than patients with non-adenocarcinoma (54/2724; 2.0%) (OR = 2.25; 95% CI: 1.54–3.27; P<0.0001; *I* ^2^ = 26%). In subgroup analysis by race, significance was only noted in Asians (OR = 2.92; 95% CI: 1.88–4.53; P<0.00001), without heterogeneity (*I* ^2^ = 14%) or publication bias (N_fs_ = 249.5) (Figure S7). Compared with squamous carcinomas, lung adenocarcinomas was associated with an increased rate of ALK rearrangements in Asians (OR = 3.64; 95% CI: 2.17–6.09; P<0.00001) with no heterogeneity (*I* ^2^ = 0%) and publication bias (N_fs_ = 168.2), whereas this increase was not obvious in Caucasians (OR = 1.33; 95% CI: 0.48–3.68; P = 0.58; *I* ^2^ = 0%) with evident publication bias (N_fs_ = −0.83) (Figure S8).

### ALK rearrangements and EGFR/KRAS mutations

Generally, EGFR/KRAS mutations were more common than ALK rearrangements in NSCLC patients, and they rarely coexisted according to available reports in Table S4. Never-smokers tended to have EGFR mutations and ALK rearrangements, and smokers more likely had KRAS mutations. Moreover, ALK rearrangements tended to occur in younger NSCLC patients compared with EGFR (Figure S9) and KRAS (Figure S10) mutations, and there was no observable publication bias.

### Meta-regression analysis

To further explore the extent to which study-level variables explain heterogeneity among individual effect estimates, we performed a set of meta-regression analyses, and none of clinicopathological or demographical characteristics examined can significantly explain the changes of ALK rearrangements in NSCLC patients (data not shown).

## Discussion

Via a meta-analysis of the data from 62 articles and on 20541 NSCLC patients, we examined the relationship between a panel of clinicopathological and demographical characteristics and ALK rearrangements. The most notable finding of this study was that ALK rearrangements tended to be present in NSCLC patients with no smoking habit, younger age and tumor stage IV. Moreover, race, age, gender, smoking status, tumor stage and histology might be potential sources of between-study heterogeneity. Although other sources of heterogeneity cannot be easily ruled out, this study, to the best of our knowledge, is so far the largest meta-analysis examining the relationship between clinicopathological or demographical characteristics and ALK rearrangements in NSCLC patients.

A substantial body of evidence supports the prognostic and predictive value of ALK rearrangements in NSCLC patients [75]. Given the low prevalence of ALK rearrangements in NSCLC patients, the present meta-analysis was designed to resolve inconsistent results based on sparse data on clinicopathological or demographical characteristics from studies that may have been inconclusive due to the small sample size involved. A recent meta-analysis by Li and colleagues in 14 articles involving 125 ALK rearranged cases from 2580 NSCLC patients indicated high rate of ALK rearrangements in never-smokers with adenocarcinomas [50]. The present study is more comprehensive than the study by Li and colleagues in terms of the following aspects: first, we focused on a broader spectrum of clinicopathological or demographical characteristics in relation with ALK rearrangements in NSCLC patients; second, a more comprehensive subgroup analyses were conducted by age, gender, race, smoking status, tumor stage and histology, the existence of EGFR/KRAS mutations; third, our study involved more relevant articles (62 qualified articles: 1178 ALK rearranged cases from 20541 NSCLC patients), as the large number of articles provided sufficient power to assess a modest effect estimate. Consistent with the results of study by Li and colleagues and others [30], [50], never-smokers had a low occurrence rate of ALK rearrangements compared with smokers among NSCLC patients. In case of gender, conflicting findings were reported [9], [11], [38], [69], as well as in the meta-analysis by Li and colleagues [50], whereas our data showed remarkably lower risk of carrying ALK rearrangements in males than females.

Besides the smoking status- and gender-specific differences in clinicopathological or demographical features between NSCLC patients with and without ALK rearrangements, ethnic difference merits special consideration. Previous studies found similar overall distributions of ALK rearrangements between NSCLC patients of Asian and Caucasian descent [43], [76]. However in this meta-analysis, the prevalence of ALK rearrangements was heterogeneous between East Asians and Caucasians in terms of age, gender and histology. For example, ALK rearrangements tended to be prevalent in female and non-squamous patients in East Asians; however, there were no obvious clinical differences in Caucasians, indicating that tumorigenesis may be at least in part explained by race. As such, the findings presented in this meta-analysis must be evaluated with caution, as there is a danger of extrapolating the findings of Asians to other ethnic groups with high prevalence of ALK rearrangements.

Our findings also confirmed the view that ALK rearrangements are more common in patients at advanced NSCLC [69], [72] and in patients with lung adenocarcinoma than with non-adenocarcinoma especially squamous cell carcinoma [8], [9]. In agreement with the results of most previous reports, our meta-analysis indicated that ALK rearrangements were most frequently occurred in NSCLC patients of stage IV. Additionally extending this view, we in this meta-analysis found that the relationship between ALK rearrangements and lung cancer histology was race-dependent. In NSCLC patients of East Asian descent, lung adenocarcinomas had a markedly higher rate of ALK rearrangements than squamous cell carcinomas, while this was not the case in Caucasians, likely due to more cases of squamous cell carcinoma in Caucasians than East Asians (2.1% versus 0.8%).

Except for ALK rearrangements, other mutations such as EGFR/KRAS mutations were also commonly seen in NSCLC patients [43], [44]. However, there were rare coexistences of ALK rearrangements with EGFR/KRAS mutations. Considering that a considerable proportion of NSCLC patients had EGFR/KRAS mutations, it could be expected that ALK rearrangements will be more common among patients with EGFR/KRAS wild-type mutations, as reported by some observations [60], [62], [77]. It is of interest to note that Asian NSCLC patients who had ALK rearrangements shared similar features to those with EGFR mutations in terms of gender, smoking status and adenocarcinoma [1]. Therefore, improved understanding of EGFR/KRAS mutation status may facilitate the identification of NSCLC patients with ALK rearrangements, and further the development of more targeted therapy.

Some limitations should be considered when interpreting our results. First, only published studies in English language were retrieved in this meta-analysis, and publication bias might be possible. Second, ALK rearrangements were rare in NSCLC patients, which may limit the statistical power to detect publication bias. Third, the detection methods of ALK rearrangements were heterogeneous across retrieved articles, which may increase the risk of between-study heterogeneity. Last but not the least, there were many other clinicopathological or demographical characteristics that were poorly known, such as high frequencies of signet-ring cell and mucinous cribriform patterns in ALK rearranged adenocarcinomas [17], [78].

Taken together, our findings demonstrate that ALK rearrangements tended to be present in NSCLC patients with no smoking habit, younger age and tumor stage IV. Moreover, race, gender, age, smoking status, tumor stage and histology might be potential sources of between-study heterogeneity. Nevertheless, for practical reasons, we hope that this study will not remain just another endpoint of research but instead would encourage more validation studies of our findings in other independent large populations, which would acquiring a better understanding of ALK rearrangements in NSCLC patients.

## Supporting Information

Checklist S1 **The PRISMA checklist.**
(DOC)

Figure S1 **Forest plots of the mean difference of age between lung adenocarcinomas patients with and without ALK rearrangements by race.**
(PDF)

Figure S2 **Forest plots of gender difference between lung adenocarcinomas patients with and without ALK rearrangements by race.**
(PDF)

Figure S3 **Forest plots of smoking difference between NSCLC patients with and without ALK rearrangements by race.**
(PDF)

Figure S4 **Forest plots of smoking difference between lung adenocarcinomas patients with and without ALK rearrangements by race.**
(PDF)

Figure S5 **Forest plots of tumor stage difference between NSCLC patients with and without ALK rearrangements by race.**
(PDF)

Figure S6 **Forest plots of tumor stage difference between lung adenocarcinomas patients with and without ALK rearrangements by race.**
(PDF)

Figure S7 **Forest plots of distribution difference in ALK rearrangements between patients with and without lung adenocarcinomas by race.**
(PDF)

Figure S8 **Forest plots of distribution difference in ALK rearrangements between patients with lung adenocarcinomas and squamous carcinomas by race.**
(PDF)

Figure S9 **Forest plots of the mean difference of age between NSCLC patients with ALK rearrangements and with EGFR mutations by race.**
(PDF)

Figure S10 **Forest plots of the mean difference of age between NSCLC patients with ALK rearrangements and with KRAS mutations by race.**
(PDF)

Table S1 **The baseline characteristics of all qualified studies in this meta-analysis.**
(DOC)

Table S2 **ALK rearrangements and tumor stage.**
(DOC)

Table S3 **ALK rearrangements and NSCLC histology.**
(DOC)

Table S4 **The baseline characteristics of all qualified articles assessing both ALK rearrangements and EGFR/KRAS mutations.**
(DOC)
