# Supplementary figures and images for: Clinicopathological and Demographical Characteristics of Non-Small Cell Lung Cancer Patients with ALK Rearrangements: A Systematic Review and Meta-Analysis
Source: PLoS One. 2014 Jun 24;9(6):e100866. doi: 10.1371/journal.pone.0100866 (PMC4069179; doi:10.1371/journal.pone.0100866)

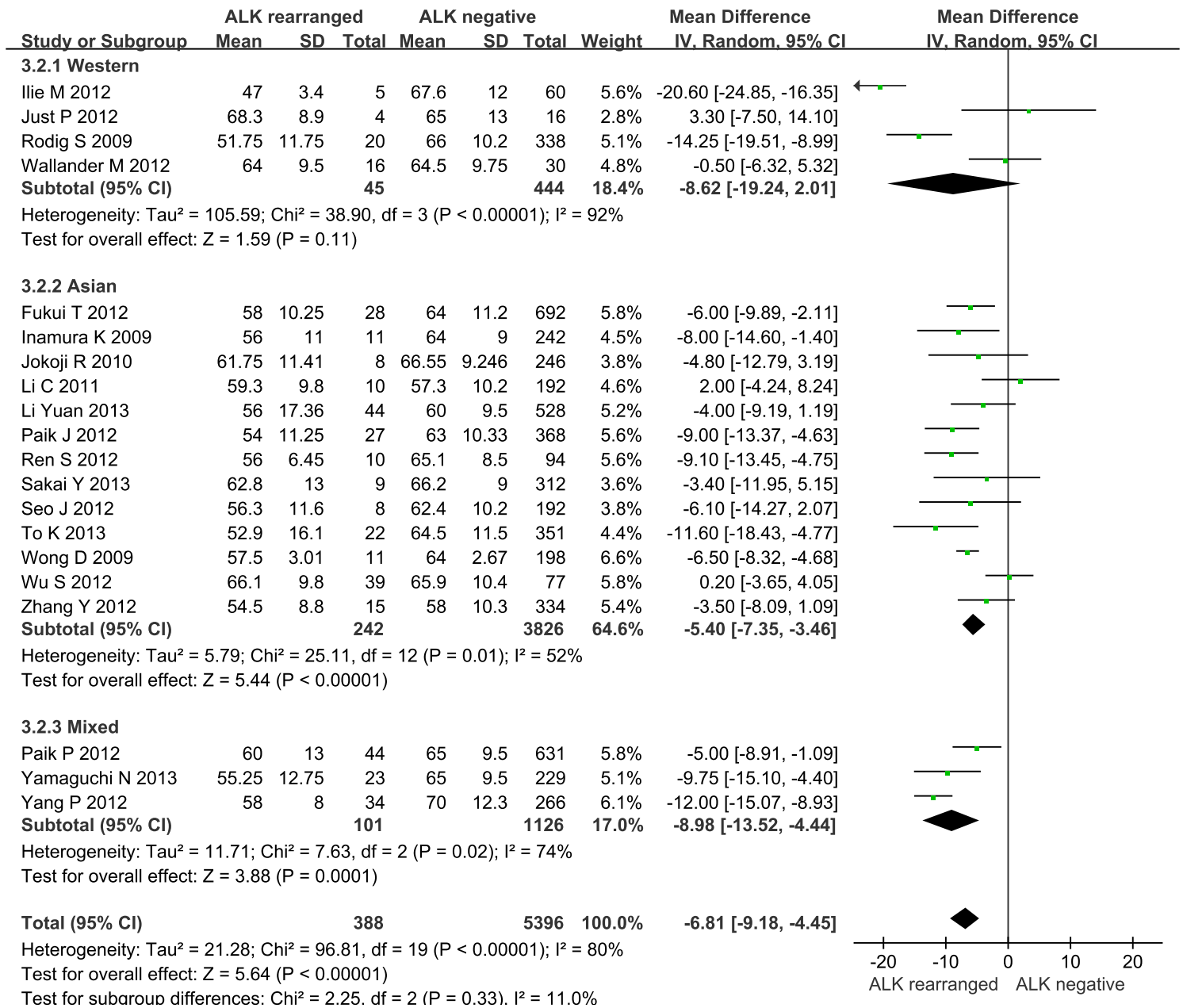

Supplement: Figure S1 — Forest plots of the mean difference of age between lung adenocarcinomas patients with and without ALK rearrangements by race. (PDF) [file pone.0100866.s002.pdf]

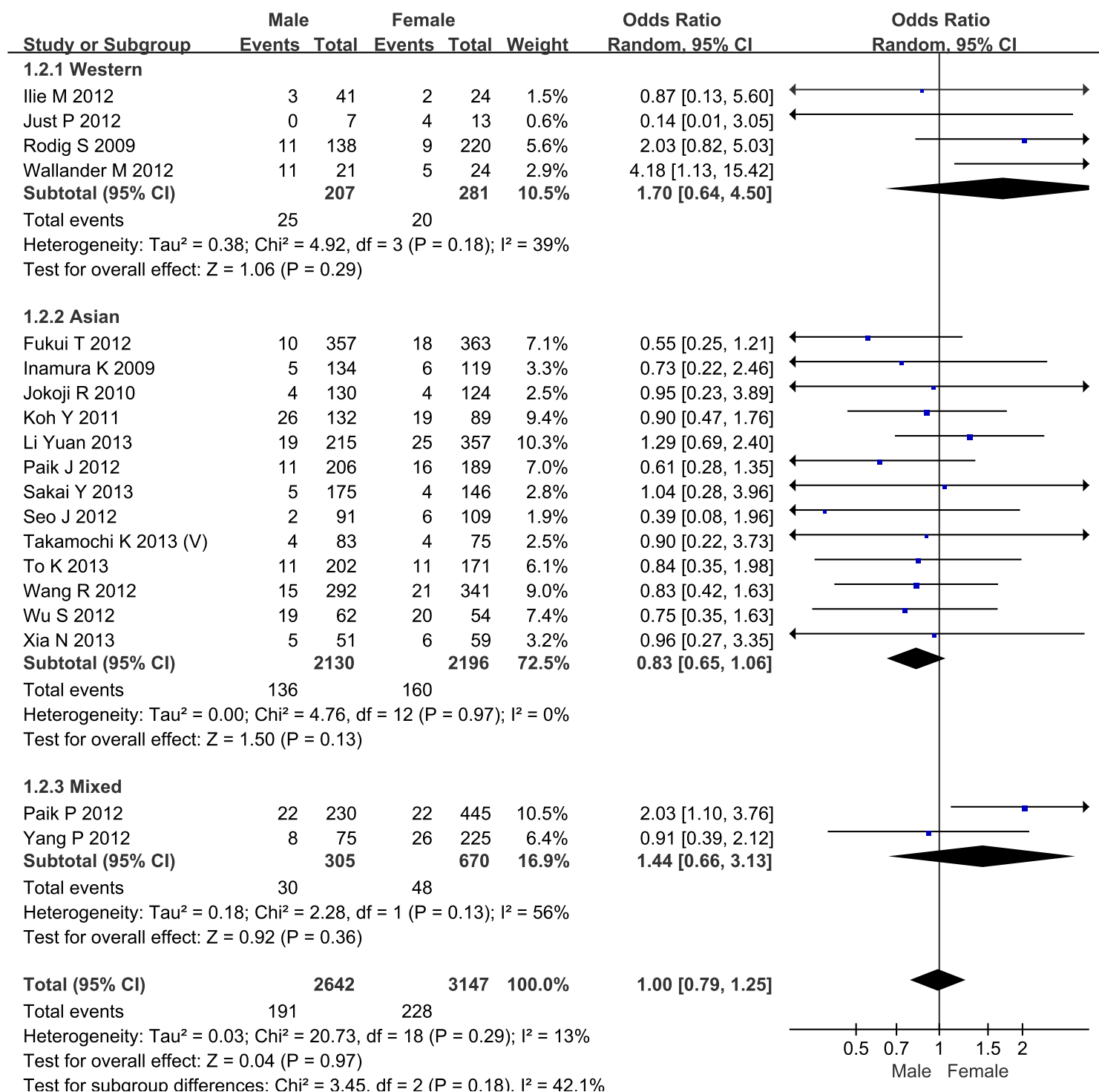

Supplement: Figure S2 — Forest plots of gender difference between lung adenocarcinomas patients with and without ALK rearrangements by race. (PDF) [file pone.0100866.s003.pdf]

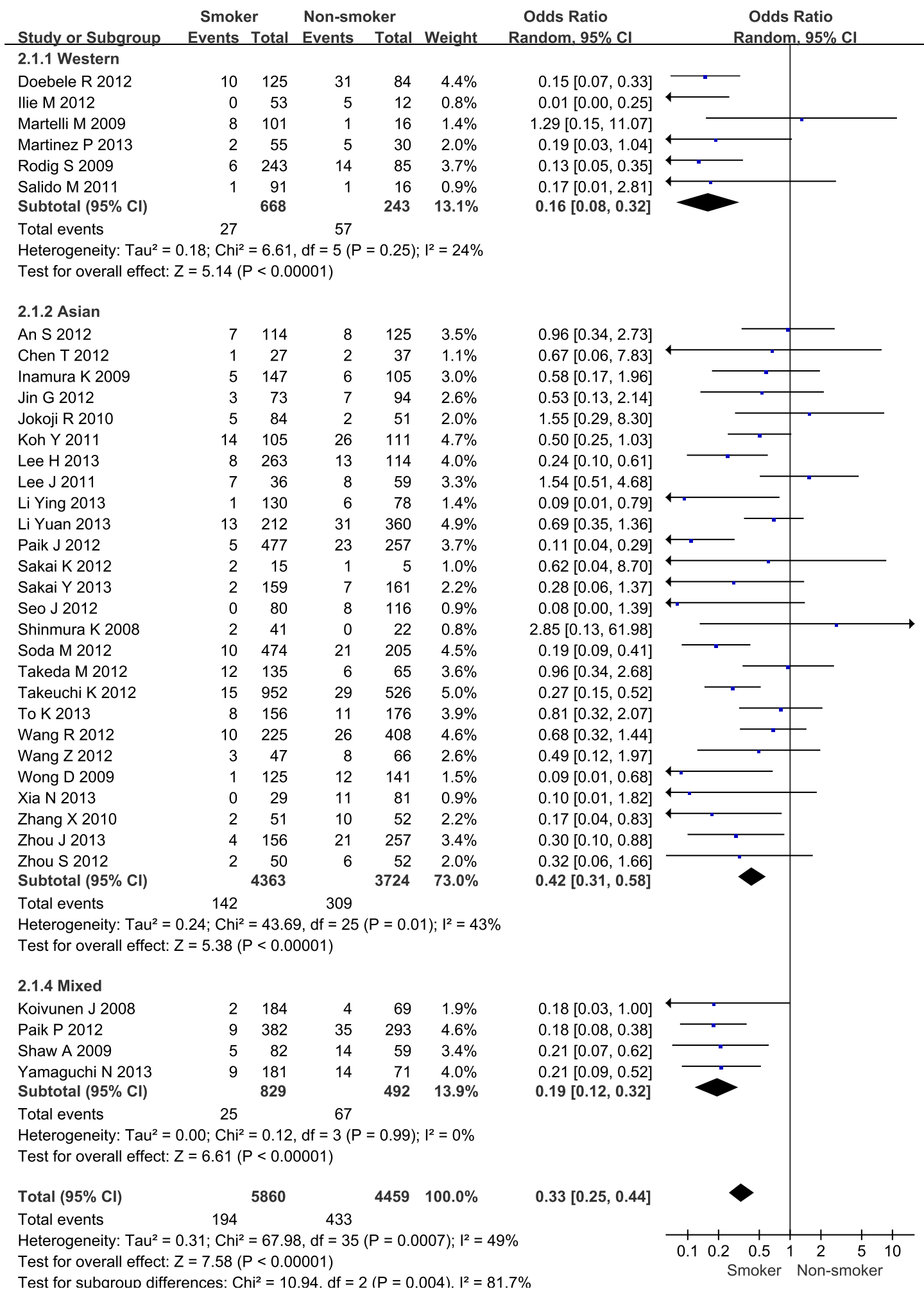

Supplement: Figure S3 — Forest plots of smoking difference between NSCLC patients with and without ALK rearrangements by race. (PDF) [file pone.0100866.s004.pdf]

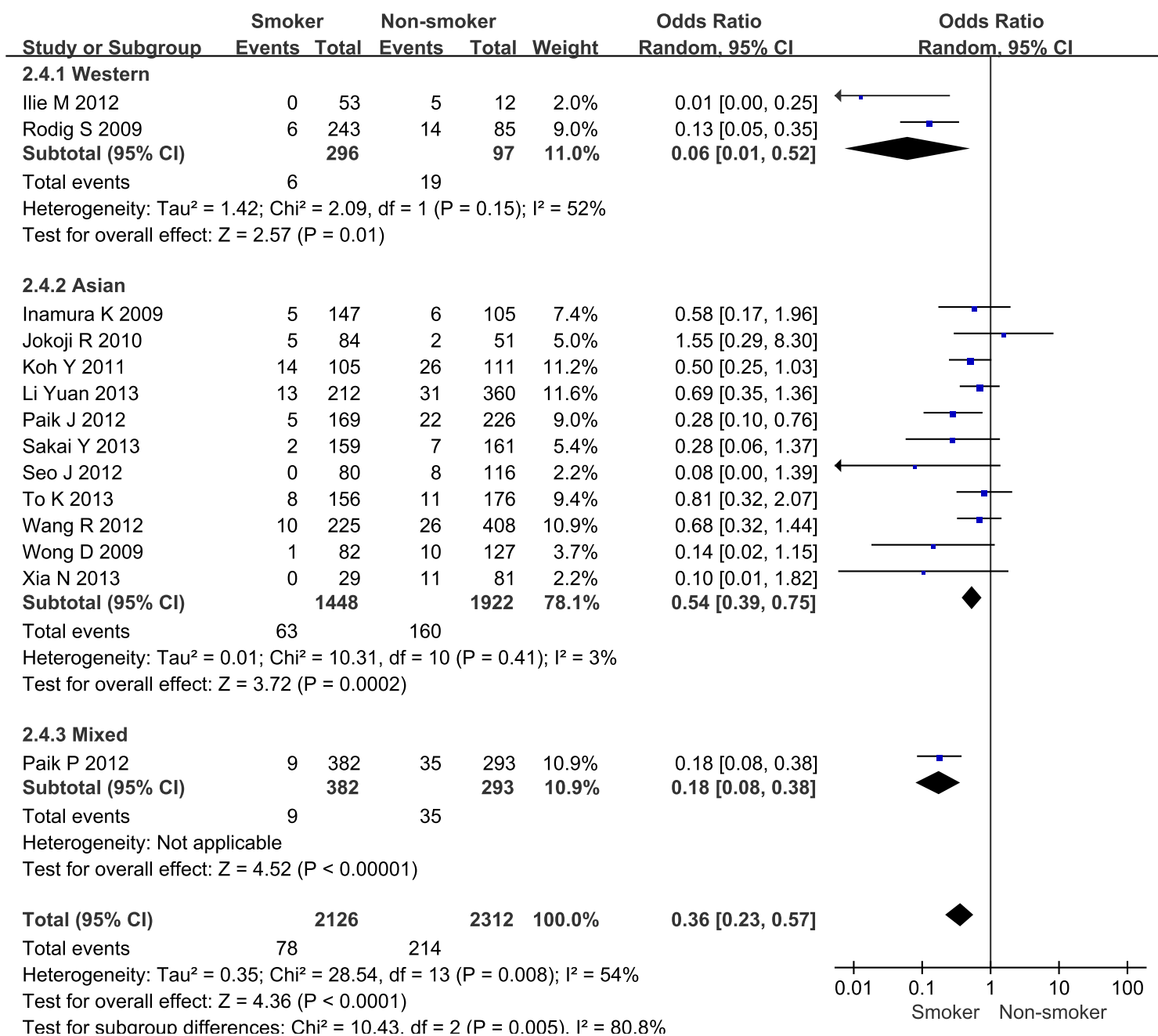

Supplement: Figure S4 — Forest plots of smoking difference between lung adenocarcinomas patients with and without ALK rearrangements by race. (PDF) [file pone.0100866.s005.pdf]

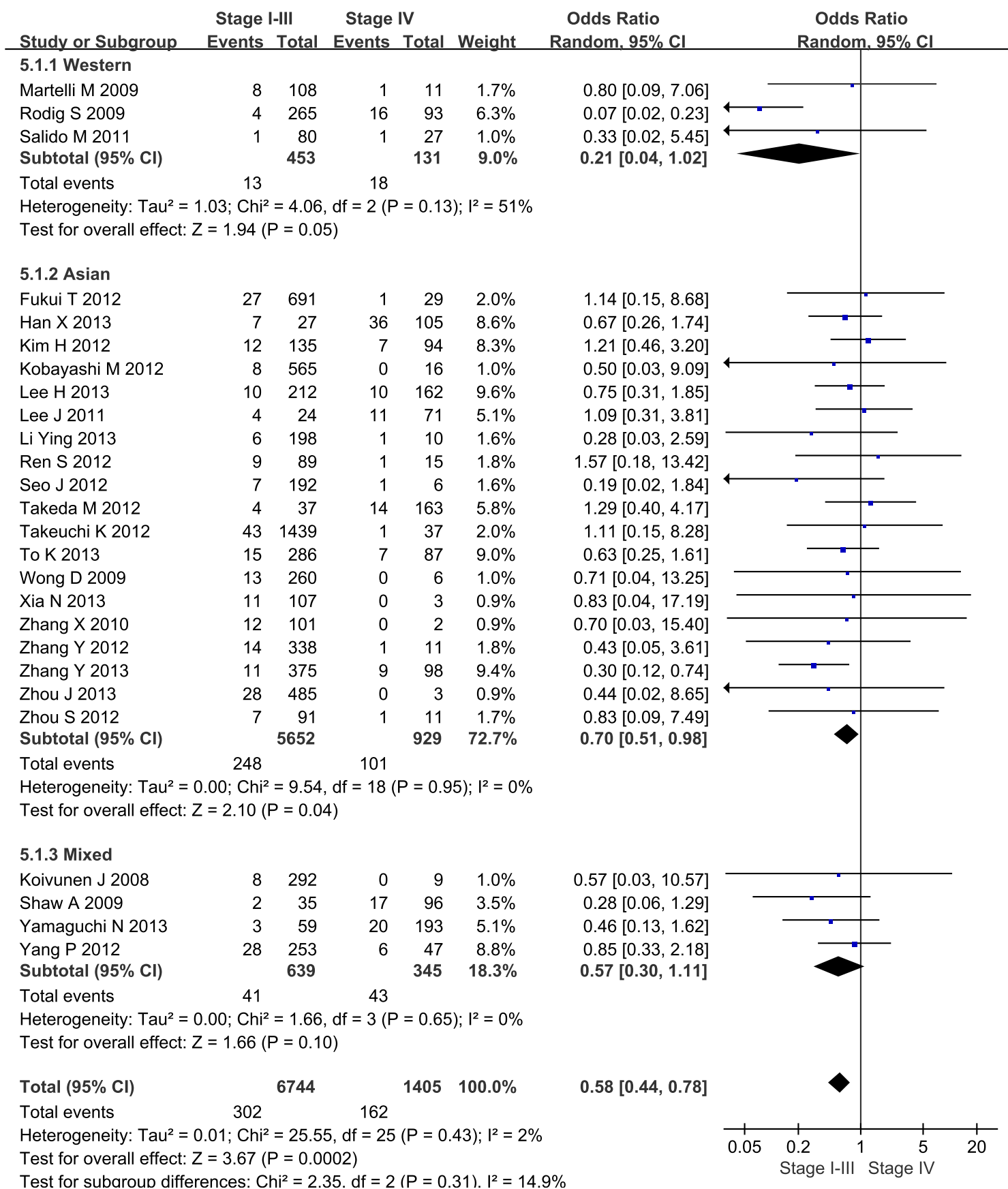

Supplement: Figure S5 — Forest plots of tumor stage difference between NSCLC patients with and without ALK rearrangements by race. (PDF) [file pone.0100866.s006.pdf]

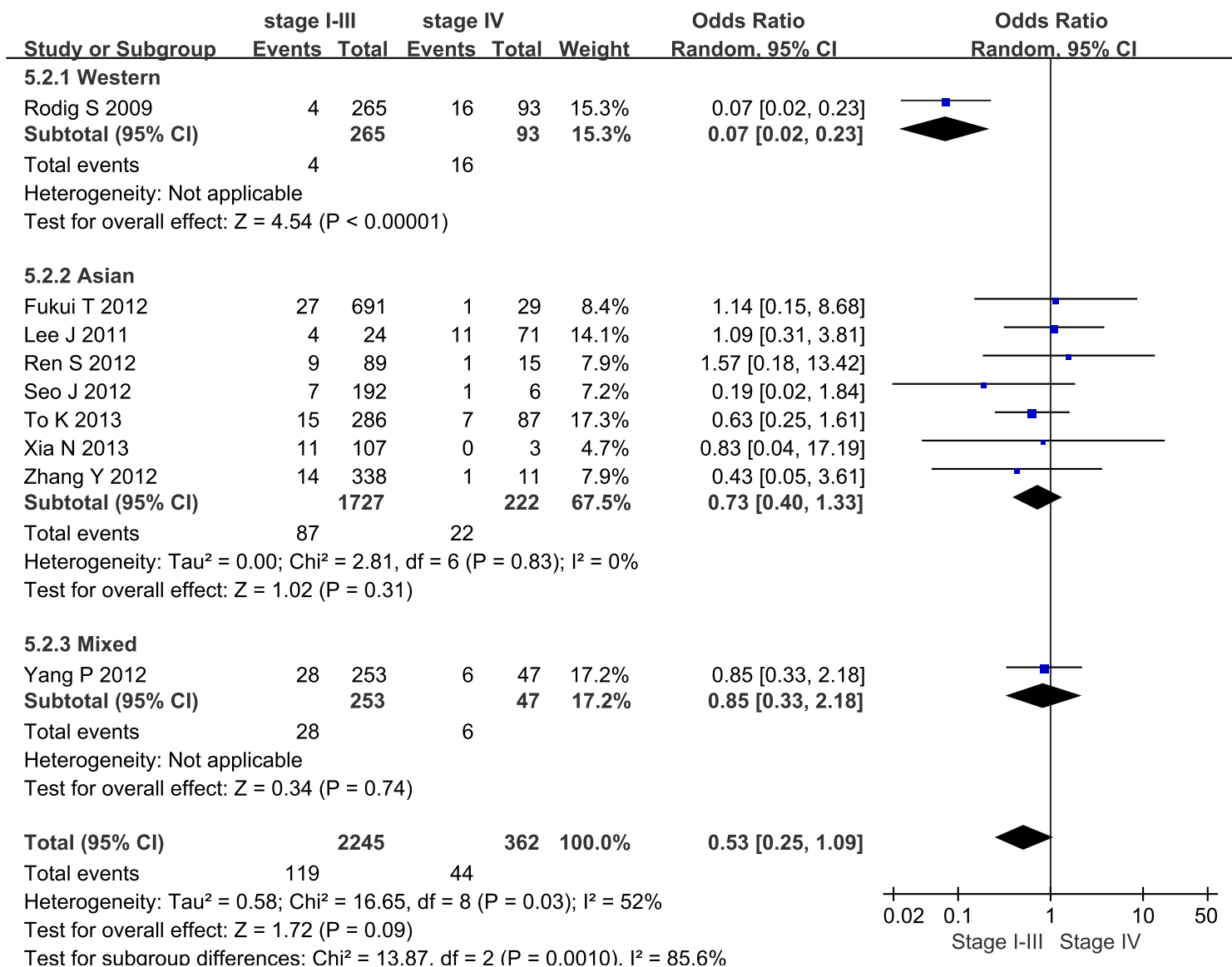

Supplement: Figure S6 — Forest plots of tumor stage difference between lung adenocarcinomas patients with and without ALK rearrangements by race. (PDF) [file pone.0100866.s007.pdf]

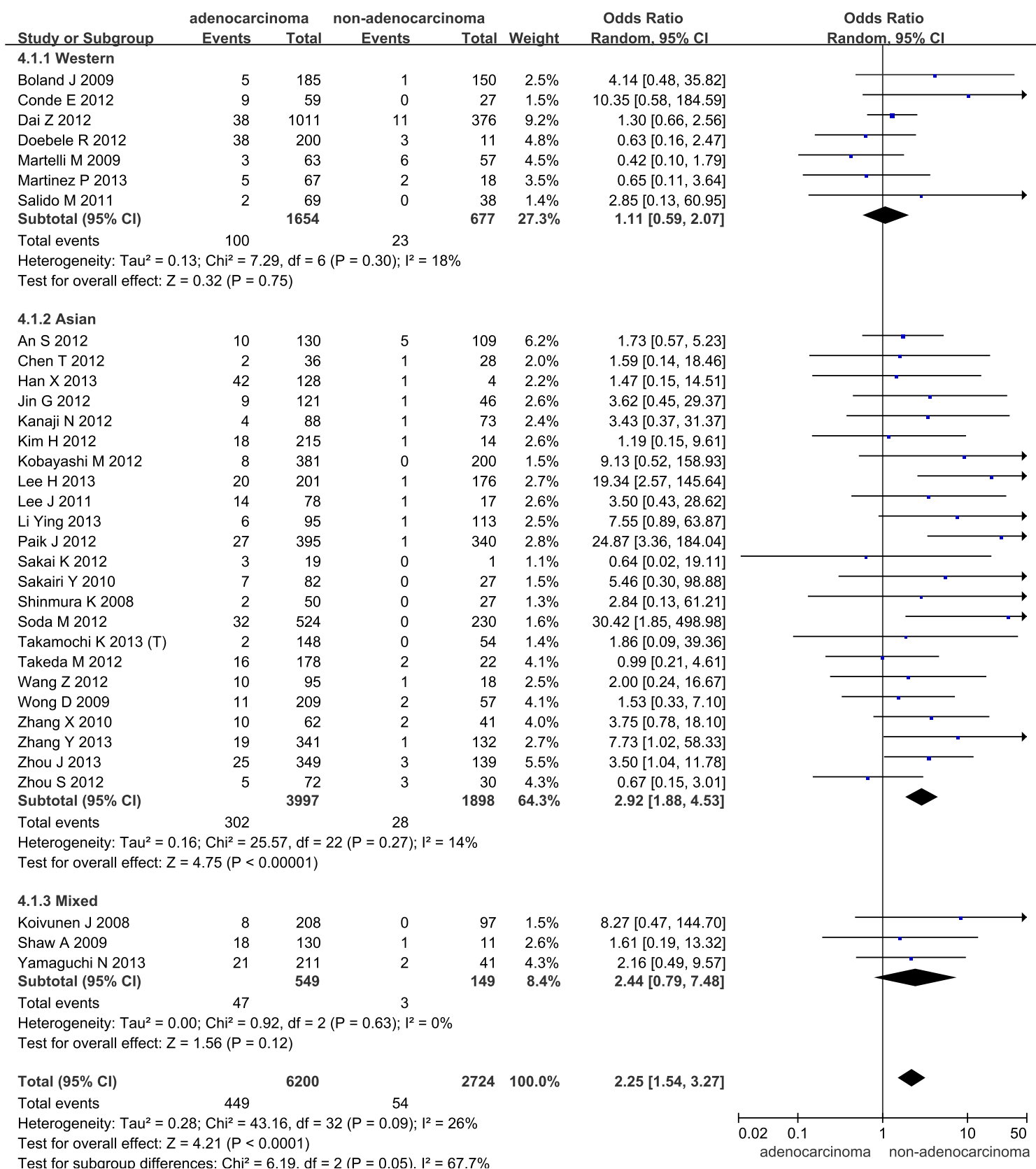

Supplement: Figure S7 — Forest plots of distribution difference in ALK rearrangements between patients with and without lung adenocarcinomas by race. (PDF) [file pone.0100866.s008.pdf]

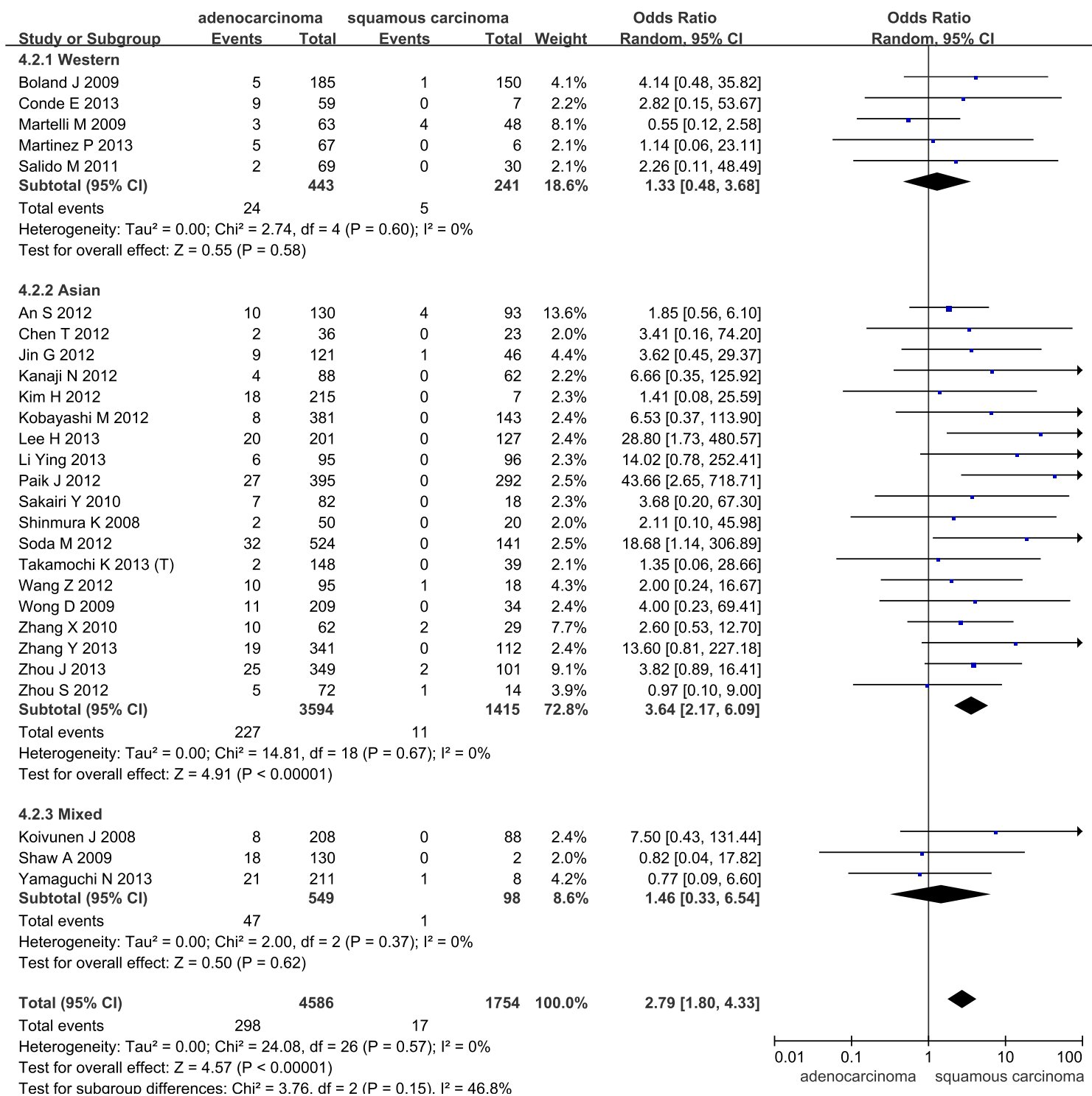

Supplement: Figure S8 — Forest plots of distribution difference in ALK rearrangements between patients with lung adenocarcinomas and squamous carcinomas by race. (PDF) [file pone.0100866.s009.pdf]

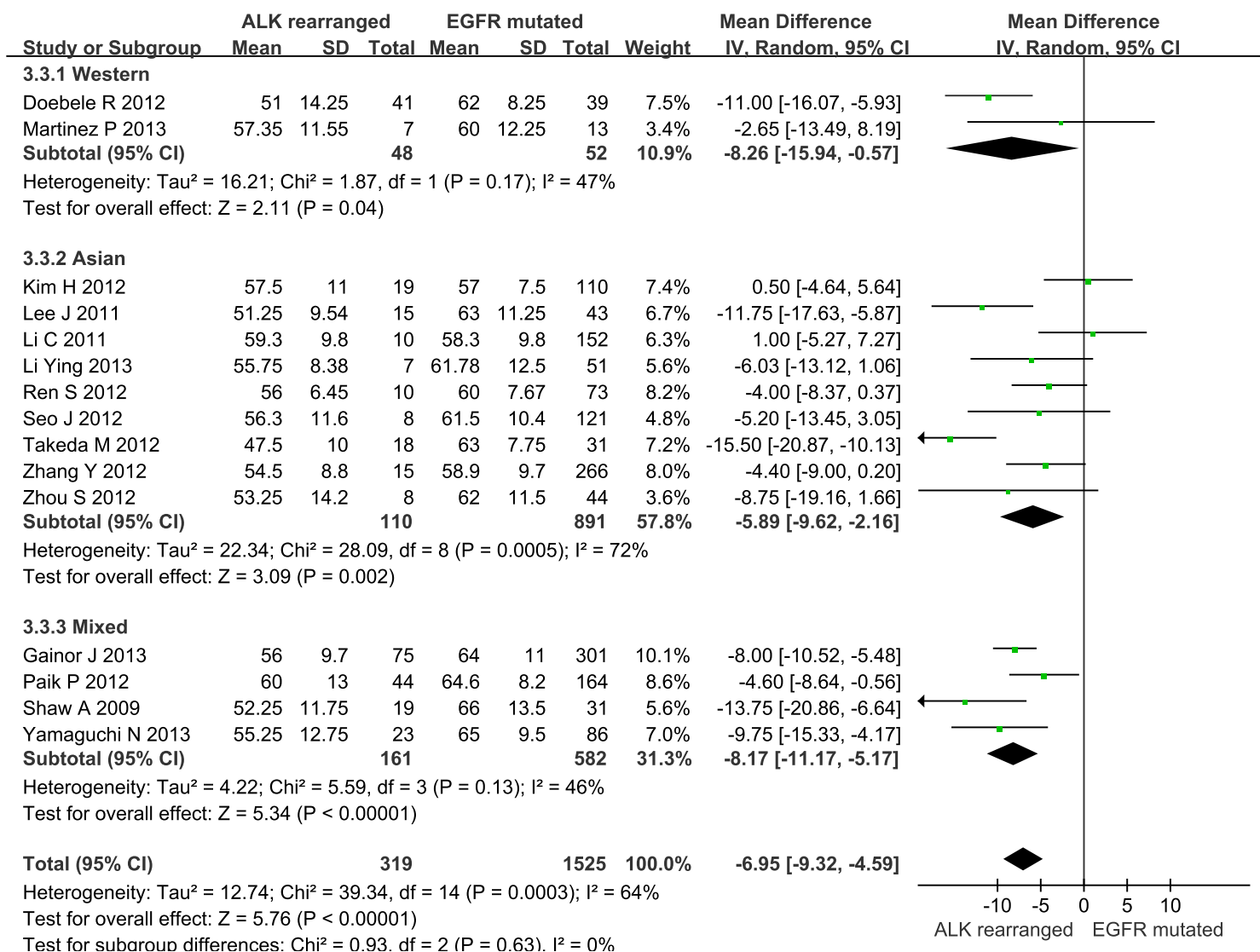

Supplement: Figure S9 — Forest plots of the mean difference of age between NSCLC patients with ALK rearrangements and with EGFR mutations by race. (PDF) [file pone.0100866.s010.pdf]

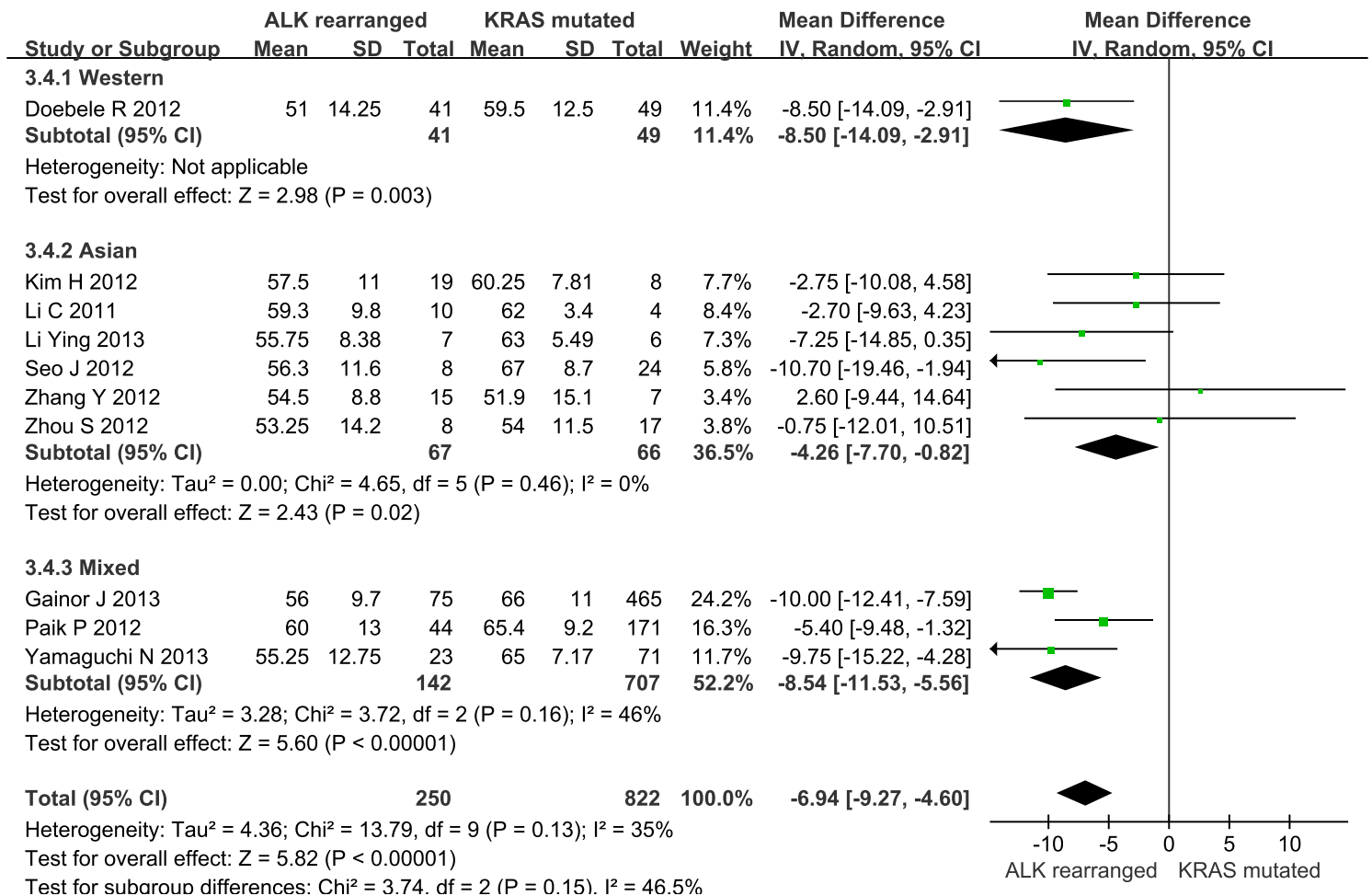

Supplement: Figure S10 — Forest plots of the mean difference of age between NSCLC patients with ALK rearrangements and with KRAS mutations by race. (PDF) [file pone.0100866.s011.pdf]
