# Supplementary material for: Clinicopathological and Demographical Characteristics of Non-Small Cell Lung Cancer Patients with ALK Rearrangements: A Systematic Review and Meta-Analysis
Source: PLoS One. 2014 Jun 24;9(6):e100866. doi: 10.1371/journal.pone.0100866 (PMC4069179; doi:10.1371/journal.pone.0100866)
Supplement: Table S1 — The baseline characteristics of all qualified studies in this meta-analysis. (DOC) [file pone.0100866.s012.doc]

**Table S1. The baseline characteristics of all included studies in this meta-analysis**

**Table S1 (A). Articles in unselected populations**

| **Reference** | **Ethnicity** | **Method** | **Histology** | **ALK^+^/Total** | **Gender** | | **Smoking****-status** | | **Age** | | **Characteristics** |
| --- | --- | --- | --- | --- | --- | --- | --- | --- | --- | --- | --- |
|  |  |  |  |  | Male | Female | Never | Ever | ALK+ | Wild type |  |
| Zhou J 2013 | Chinese | RT-PCR | NSCLC | 28/488 | 9/295 | 19/193 | 21/257 | 4/156 | NA | NA | 75 patients with unknown smoking habit |
| Wang R 2012 | Chinese | RT-PCR and FISH | Ad | 36/633 | 15/292 | 21/341 | 26/408 | 10/225 | NA | NA | / |
| Dai Z 2012 | American | FISH and RT-PCR | NSCLC | 49/1387 | NA | NA | NA | NA | NA | NA | / |
| Conde E 2013 | [European](app:ds:European) | FISH | NSCLC | 9/86 | 1/49 | 8/37 | NA | NA | 51.33±17.38 | 60.34±10.12 | / |
| Cardarella 2012 | Mixed | FISH or IHC | NSCLC | 16/309 | NA | NA | NA | NA | 54(24–79) | NA | mostly White, non-Hispanic |
| Seo J 2012 | Korean | FISH | Ad | 8/200 | 2/91 | 6/109 | 8/116 | 0/80 | 56.3±11.6 | 62.4±10.2 | / |
| Takeuchi K 2012 | Japanese | IHC and RT-PCT | NSCLC | 44/1485 | 19/854 | 25/631 | 29/526 | 15/952 | 59(26-84) | 65(25-86) | 41 EML4-ALK and 3 KIF5B-ALK rearrangements |
| Soda M 2012 | Japanese | RT-PCR | NSCLC | 32/754 | 10/492 | 22/262 | 21/205 | 10/474 | 48.3±15.9 | 65.5±12.2 | / |
| Sakai K 2012 | Japanese | PCR and FISH | NSCLC | 3/20 | 2/14 | 1/6 | 1/5 | 2/15 | 62.7±2.5 | 67.1±7.7 | Use the MassARRAY iPLEX platform for detection of EML4-ALK rearrangements. |
| Rimkunas 2012 | Chinese | IHC and FISH | NSCLC | 22/556 | NA | NA | NA | NA | NA | NA | / |
| Paik P 2012 | Mixed | FISH | Ad | 44/675 | 22/230 | 22/445 | 35/293 | 9/382 | 60(32-84) | 65(31-88) | Mostly white (579/675) |
| Paik J 2012 | Korean | FISH | NSCLC | 28/735 | 11/510 | 17/225 | 23/257 | 5/477 | 55(35–80) | 64(20–84) | / |
| Lee J 2012 | Korean | FISH | Ad | 34/444 | NA | NA | NA | NA | NA | NA | / |
| Kanaji N 2012 | Japanese | RT-PCR | NSCLC | 5/161 | NA | NA | NA | NA | 58 | NA | Transbronchial cytological specimens |
| Jin G 2012 | Korean | RT-PCR | NSCLC | 10/167 | 4/85 | 6/82 | 7/94 | 3/73 | NA | NA | / |
| Ilie M 2012 | Caucasian | FISH and IHC | Ad | 5/65 | 3/41 | 2/24 | 5/12 | 0/53 | 47±3.4 | 67.6(37-85) | Patients with circulating tumor cells |
| Fukui T 2012 | Japanese | RT-PCR and IHC | Ad | 28/720 | 10/357 | 18/363 | NA | NA | 58(35-76) | 64(22-89) | <20 pack-years (22/436), ≥20 pack-years (6/284) |
| Chen T 2012 | Chinese | RT-PCR and IHC | NSCLC | 3/64 | 1/40 | 2/24 | 2/37 | 1/27 | 62.5(50-67) | 65(29-84) | / |
| An S 2012 | Chinese | PCR | NSCLC | 15/239 | NA | NA | 8/125 | 7/114 | NA | NA | / |
| Salido M 2011 | [Spanish](app:ds:Spanish) | FISH | NSCLC | 2/107 | 1/82 | 1/25 | 1/16 | 1/91 | 73±1.4 | 66(40-85) | / |
| Zhang X 2010 | Chinese | RT-PCR | NSCLC | 12/103 | 7/74 | 5/29 | 10/52 | 2/51 | 53 | 61 | / |
| Sakairi Y 2010 | Japanese | IHC | NSCLC | 7/109 | 3/82 | 4/27 | NA | NA | 55.4 | 65.0 | EBUS-TBNA specimens, never + light smokers 6/37, heavy smokers 1/70. (Smoking history was recorded in 107 patients). |
| Jokoji R 2010 | Japanese | IHC | Ad | 8/254 | 4/130 | 4/124 | 2/51 | 5/84 | 61.75±11.41 | 66.55±9.246 | 119 patients with unknown smoking habit |
| Wong D 2009 | Chinese | RT-PCR | NSCLC | 13/266 | 5/132 | 8/134 | 12/141 | 1/125 | 59(51.5-64.5) | 64(55-71) | / |
| Rodig S 2009 | Caucasian | IHC and FISH | Ad | 20/358 | 11/138 | 9/220 | 14/85 | 6/243 | 51(29-76) | 66(29-90) | 30 patients with unknown smoking habit |
| Martelli M 2009 | European | FISH and RT-PCR | NSCLC | 9/120 | 8/96 | 1/24 | 1/16 | 8/101 | 64 | 67 | / |
| Inamura K 2009 | Japanese | RT-PCR and FISH | Ad | 11/253 | 5/134 | 6/119 | 6/105 | 5/147 | 56±11 | 64±9 | / |
| Boland J 2009 | American | IHC | NSCLC | 6/335 | NA | NA | NA | NA | 69.8 | 69.6 | / |
| Shinmura K 2008 | Japanese | RT-PCR | NSCLC | 2/77 | 1/39 | 1/38 | 0/22 | 2/41 | 53 | 64.6 | / |
| Perner S 2008 | Caucasian | FISH | NSCLC | 16/603 | NA | NA | NA | NA | NA | NA | / |
| Koivunen J 2008 | Mixed | RT-PCR and FISH | NSCLC | 8/305 | NA | NA | 4/69 | 2/184 | 55.9 | 61.9 | U.S. cohort 2/138, Korean cohort 6/167 |
| Zhang Y 2013 | Chinese | FISH, IHC, RT-PCR | NSCLC | 20/473 | 14/314 | 6/159 | NA | NA | 44.8±16.2 | 59.6±10.5 | Non- or Iight smoker (<10 pack year) 7/180, smoker (>10 pack year) 13/293 |
| Yamaguchi N 2013 | Mixed | FISH | NSCLC | 23/252 | 12/98 | 11/154 | 14/71 | 9/181 | 56(29–80) | 65(33–90) | 75.9% patients White race |
| Xia N 2013 | Chinese | FISH and RT-PCR | Ad | 11/110 | 5/51 | 6/59 | 11/81 | 0/29 | NA | NA | / |
| To K 2013 | Chinese | FISH, IHC, RT-PCR | Ad | 22/373 | 11/202 | 11/171 | 11/176 | 8/156 | 52.9±16.1 | 64.5±11.5 | / |
| Takamochi K 2013 | Japanese | FISH, IHC, RT-PCR | NSCLC | 2/202 | 0/122 | 2/80 | 1/84 | 1/118 | 56.5 | NA | Test set; Never smoker: pack-year≤5. |
| Sakai Y 2013 | Japanese | IHC and FISH | Ad | 9/321 | 5/175 | 4/146 | 7/161 | 2/159 | 62.8±13.0 | 66.2±9.0 | / |
| Martinez P 2013 | Caucasian | FISH, IHC | NSCLC | 7/85 | 3/42 | 4/43 | 5/30 | 2/55 | 56.7(38-78) | 62(36-83) | / |
| Li Yuan 2013 | Chinese | FISH, IHC, RT-PCR | Ad | 44/572 | 19/215 | 25/357 | 31/360 | 13/212 | 56(22-82) | 60(27-84) | / |
| Li Ying 2013 | Chinese | FISH and RT-PCR | NSCLC | 7/208 | 0/147 | 7/61 | 6/78 | 1/130 | 55±10.38 | 61.78±8.71 | / |
| Lee H 2013 | Korean | IHC and FISH | NSCLC | 21/377 | 11/283 | 10/94 | 13/114 | 8/263 | 50(35-72) | 62(35-83) | / |
| Gainor J 2013 | Mixed | FISH | NSCLC | 75/1683 | NA | NA | NA | NA | 56(29-87) | NA | Mostly Caucasian |
| Desai 2013 | Indian | FISH | NSCLC | 5/187 | 2/141 | 3/46 | NA | NA | 46(35-61) | 54(21-79) | 5 positive, 182 negative, and 37 uninterpretable results |

**Table S1 (B). Articles enrolled specific groups of patients according to their clinical pathological characteristics or genetic makeup**

| **Reference** | **Ethnicity** | **Method** | **Histology** | **ALK^+^/Total** | **Gender** **(ALK+/Total)** | | **Smoke-status (ALK+/Total)** | | **Age (Mean or Median)** | | **Characteristics** |
| --- | --- | --- | --- | --- | --- | --- | --- | --- | --- | --- | --- |
|  |  |  |  |  | Male | Female | nonsmoker | Smoker | Mutant | Wild type |  |
| Zhang Y 2012 | Chinese | RT-PCR | Ad | 15/349 | / | 15/349 | 15/349 | / | 54.5±8.8 | 58.0±10.3 | Females, never smokers, Ad |
| Yang P 2012 | Mixed | IHC and FISH | Ad | 34/300 | 8/75 | 26/225 | 34/300 | / | 58(36-84) | 70(17-91) | Never smokers, Ad, 263/300 Caucasians |
| Yoon H 2012 | Korean | IHC and FISH | NSCLC | 11/40 | NA | NA | NA | NA | NA | NA | Resistant to conventional chemotherapy or EGFR-TKIs |
| Wu S 2012 | Chinese | RT-PCR | Ad | 39/116 | 19/62 | 20/54 | NA | NA | 66.1(27.9-86.7) | 65.9(28.8-91.1) | Malignant pleural fluid samples, EGFR wild-type. Non- or Iight smoker <10 pack-years (29/81), smoker ≥10 pack-years (10/35) |
| Wang Z 2012 | Chinese | IHC and FISH | NSCLC | 11/113 | 4/58 | 7/55 | 8/66 | 3/47 | 62(45-72) | 60(22-85) | Stage IV |
| Wallander M 2012 | American | FISH and RT-PCR | Ad | 16/46 | 11/21 | 5/24 | NA | NA | 65(44-82) | 64.5(49-88) | 42 of 46 samples (91%) were EGFR wild-type |
| Takeda M 2012 ­ | Japanese | PCR | NSCLC | 18/200 | 9/127 | 9/73 | 6/65 | 12/135 | 46(29-69) | 64(35-81) | Nonsquamous, stage IIIB or IV |
| Zhou S 2012 | Chinese | RT-PCR | NSCLC | 8/102 | 2/54 | 6/48 | 6/52 | 2/50 | 51(31-80) | 59.5(26-78) | Have at least one of the following clinical characteristics: female, no or light smoking history, and adenocarcinoma histology. |
| Ren S 2012 | Chinese | RT-PCR | Ad | 10/104 | / | 10/104 | 10/104 | / | 54.0(47-69) | 65.1(27-78) | Females, never smokers |
| Park H 2012 | Korean | FISH and IHC | NSCLC | 25/262 | 11/157 | 14/105 | NA | NA | 47.6(22-63) | 64.7(22-91) | Non-squamous, EGFR wild-type or non-responders to previous EGFR-TKIs |
| Kim H 2012 | Korean | FISH and IHC | NSCLC | 19/229 | 2/30 | 17/199 | 19/229 | / | 59(34-78) | 58(30-78) | Never smokers |
| Just P 2012 | Caucasian | RT-PCR | Ad | 4/20 | 0/7 | 4/13 | NA | NA | 68.3±8.9 | 65.0±13.0 | EGFR wild-type, no or light smoking history |
| Doebele R 2012 | American | FISH | NSCLC | 41/209 | 21/83 | 20/126 | 31/84 | 10/125 | 51(21-78) | 61(32-82) | Nonsquamous, stage IV |
| Li C 2011 | Chinese | Multiplex RT-PCR | Ad | 10/202 | 4/43 | 6/159 | 202/0 | NA | 59.3±9.8 | 57.3±10.2 | Never smokers |
| Koh Y 2011 | Korean | IHC and FISH | Ad | 45/221 | 26/132 | 19/89 | 26/111 | 14/105 | 49 | 61 | Advanced adenocarcinoma either metastatic or recurrent at the time of ALK screening |
| Shaw A 2009 | Mixed | IHC and FISH | NSCLC | 19/141 | 11/48 | 8/93 | 14/59 | 5/82 | 52(29-76) | 64.7(29-90) | With two or more of the following clinical characteristics: females, Asian ethnicity, never/light smoking history, and adenocarcinoma histology. |
| Han X 2013 | Chinese | FISH, IHC, RT-PCR | NSCLC | 43/132 | 17/56 | 26/76 | NA | NA | 47.1±12.0 | 54.0±10.0 | non-squamous, stage IIIB or IV disease, previously treated with cytotoxic regimens and/or targeted therapy and had progressive disease.  Non- or Iight smoker (<10 pack year) 39/101, smoker (>10 pack year) 4/31 |
| Lee J 2011 | Korean | FISH | NSCLC | 15/95 | 7/44 | 8/51 | 8/59 | 7/36 | 52(34-67) | 59(28-79) | NSCLC at stage IIIB/IV or relapse after prior systemic chemotherapy |
| Kobayashi M 2012 | Japanese | FISH | Ad | 8/581 | 2/375 | 6/206 | NA | NA | 56.6 | 66 | Adenocarcinoma, wild-type EGFR, ERBB2, and KRAS, all non- or light smokers 8/175 (<20 packs per year), moderately or poorly differentiated |
| Takamochi K 2013 | Japanese | FISH, IHC, RT-PCR | Ad | 8/158 | 4/83 | 4/75 | 4/70 | 4/88 | 56.25 | NA | Validation set: EGFR and KRAS mutation-negative adenocarcinoma; Never smoker: pack-year≤5 |

*Abbreviations:* M/F, males/females; RT-PCR, real-time polymerase chain reaction; FISH, fluorescence *in situ* hybridization; IHC, immunehistochemistry; NSCLC, non-small-cell lung cancer; Ad, adenocarcinoma; NA, not available. Age was expressed as mean or median (year-old). Never smokers were defined as patients who had smoked <100 cigarettes in their lifetime.

**References**

1. Zhou, J.X., et al., *Oncogenic driver mutations in patients with non-small-cell lung cancer at various clinical stages.* Ann Oncol, 2013. **24**(5): p. 1319-25.

2. Wang, R., et al., *RET fusions define a unique molecular and clinicopathologic subtype of non-small-cell lung cancer.* J Clin Oncol, 2012. **30**(35): p. 4352-9.

3. Dai, Z., et al., *Incidence and patterns of ALK FISH abnormalities seen in a large unselected series of lung carcinomas.* Mol Cytogenet, 2012. **5**(1): p. 1755-8166.

4. Conde, E., et al., *The ALK translocation in advanced non-small-cell lung carcinomas: preapproval testing experience at a single cancer centre.* Histopathology, 2013. **62**(4): p. 609-16.

5. Cardarella, S., et al., *The introduction of systematic genomic testing for patients with non-small-cell lung cancer.* J Thorac Oncol, 2012. **7**(12): p. 1767-74.

6. Seo, J.S., et al., *The transcriptional landscape and mutational profile of lung adenocarcinoma.* Genome Res, 2012. **13**: p. 13.

7. Takeuchi, K., et al., *RET, ROS1 and ALK fusions in lung cancer.* Nat Med, 2012. **18**(3): p. 378-81.

8. Soda, M., et al., *A Prospective PCR-Based Screening for the EML4-ALK Oncogene in Non-Small Cell Lung Cancer.* Clin Cancer Res, 2012. **11**: p. 11.

9. Sakai, K., et al., *A novel mass spectrometry-based assay for diagnosis of EML4-ALK-positive non-small cell lung cancer.* J Thorac Oncol, 2012. **7**(5): p. 913-8.

10. Rimkunas, V.M., et al., *Analysis of receptor tyrosine kinase ROS1-positive tumors in non-small cell lung cancer: identification of a FIG-ROS1 fusion.* Clin Cancer Res, 2012. **18**(16): p. 4449-57.

11. Paik, P.K., et al., *Driver mutations determine survival in smokers and never-smokers with stage IIIB/IV lung adenocarcinomas.* Cancer, 2012. **17**(10): p. 27637.

12. Paik, J.H., et al., *Clinicopathologic implication of ALK rearrangement in surgically resected lung cancer: a proposal of diagnostic algorithm for ALK-rearranged adenocarcinoma.* Lung Cancer, 2012. **76**(3): p. 403-9.

13. Lee, J.K., et al., *Differential sensitivities to tyrosine kinase inhibitors in NSCLC harboring EGFR mutation and ALK translocation.* Lung Cancer, 2012. **77**(2): p. 460-3.

14. Kanaji, N., et al., *Detection of EML4-ALK fusion genes in a few cancer cells from transbronchial cytological specimens utilizing immediate cytology during bronchoscopy.* Lung Cancer, 2012. **77**(2): p. 293-8.

15. Jin, G., et al., *EML4-ALK fusion gene in Korean non-small cell lung cancer.* J Korean Med Sci, 2012. **27**(2): p. 228-30.

16. Ilie, M., et al., *ALK-gene rearrangement: a comparative analysis on circulating tumour cells and tumour tissue from patients with lung adenocarcinoma.* Ann Oncol, 2012. **26**: p. 26.

17. Fukui, T., et al., *Clinicoradiologic characteristics of patients with lung adenocarcinoma harboring EML4-ALK fusion oncogene.* Lung Cancer, 2012. **77**(2): p. 319-25.

18. Chen, T.D., et al., *Correlation of anaplastic lymphoma kinase overexpression and the EML4-ALK fusion gene in non-small cell lung cancer by immunohistochemical study.* Chang Gung Med J, 2012. **35**(4): p. 309-17.

19. An, S.J., et al., *Identification of enriched driver gene alterations in subgroups of non-small cell lung cancer patients based on histology and smoking status.* PLoS One, 2012. **7**(6): p. 29.

20. Salido, M., et al., *Increased ALK gene copy number and amplification are frequent in non-small cell lung cancer.* J Thorac Oncol, 2011. **6**(1): p. 21-7.

21. Zhang, X., et al., *Fusion of EML4 and ALK is associated with development of lung adenocarcinomas lacking EGFR and KRAS mutations and is correlated with ALK expression.* Mol Cancer, 2010. **9**: p. 188.

22. Sakairi, Y., et al., *EML4-ALK fusion gene assessment using metastatic lymph node samples obtained by endobronchial ultrasound-guided transbronchial needle aspiration.* Clin Cancer Res, 2010. **16**(20): p. 4938-45.

23. Jokoji, R., et al., *Combination of morphological feature analysis and immunohistochemistry is useful for screening of EML4-ALK-positive lung adenocarcinoma.* J Clin Pathol, 2010. **63**(12): p. 1066-70.

24. Wong, D.W., et al., *The EML4-ALK fusion gene is involved in various histologic types of lung cancers from nonsmokers with wild-type EGFR and KRAS.* Cancer, 2009. **115**(8): p. 1723-33.

25. Rodig, S.J., et al., *Unique clinicopathologic features characterize ALK-rearranged lung adenocarcinoma in the western population.* Clin Cancer Res, 2009. **15**(16): p. 5216-23.

26. Martelli, M.P., et al., *EML4-ALK rearrangement in non-small cell lung cancer and non-tumor lung tissues.* Am J Pathol, 2009. **174**(2): p. 661-70.

27. Inamura, K., et al., *EML4-ALK lung cancers are characterized by rare other mutations, a TTF-1 cell lineage, an acinar histology, and young onset.* Mod Pathol, 2009. **22**(4): p. 508-15.

28. Boland, J.M., et al., *Anaplastic lymphoma kinase immunoreactivity correlates with ALK gene rearrangement and transcriptional up-regulation in non-small cell lung carcinomas.* Hum Pathol, 2009. **40**(8): p. 1152-8.

29. Shinmura, K., et al., *EML4-ALK fusion transcripts, but no NPM-, TPM3-, CLTC-, ATIC-, or TFG-ALK fusion transcripts, in non-small cell lung carcinomas.* Lung Cancer, 2008. **61**(2): p. 163-9.

30. Perner, S., et al., *EML4-ALK fusion lung cancer: a rare acquired event.* Neoplasia, 2008. **10**(3): p. 298-302.

31. Koivunen, J.P., et al., *EML4-ALK fusion gene and efficacy of an ALK kinase inhibitor in lung cancer.* Clin Cancer Res, 2008. **14**(13): p. 4275-83.

32. Zhang, Y.G., et al., *Evaluation of ALK rearrangement in Chinese non-small cell lung cancer using FISH, immunohistochemistry, and real-time quantitative RT- PCR on paraffin-embedded tissues.* PLoS One, 2013. **8**(5).

33. Yamaguchi, N., et al., *Smoking status and self-reported race affect the frequency of clinically relevant oncogenic alterations in non-small-cell lung cancers at a United States-based academic medical practice.* Lung Cancer, 2013. **7**(13): p. 013.

34. Xia, N., et al., *Analysis of EGFR, EML4-ALK, KRAS, and c-MET mutations in Chinese lung adenocarcinoma patients.* Exp Lung Res, 2013. **6**: p. 6.

35. To, K.F., et al., *Detection of ALK rearrangement by immunohistochemistry in lung adenocarcinoma and the identification of a novel EML4-ALK variant.* J Thorac Oncol, 2013. **8**(7): p. 883-91.

36. Takamochi, K., et al., *A Rational Diagnostic Algorithm for the Identification of ALK Rearrangement in Lung Cancer: A Comprehensive Study of Surgically Treated Japanese Patients.* PLoS One, 2013. **8**(8).

37. Sakai, Y., et al., *Immunohistochemical Profiling of ALK Fusion Gene-Positive Adenocarcinomas of the Lung.* Int J Surg Pathol, 2013. **20**: p. 20.

38. Martinez, P., et al., *Fluorescence in situ hybridization and immunohistochemistry as diagnostic methods for ALK positive non-small cell lung cancer patients.* PLoS One, 2013. **8**(1): p. 24.

39. Li, Y., et al., *ALK-Rearranged Lung Cancer in Chinese: A Comprehensive Assessment of Clinicopathology, IHC, FISH and RT-PCR.* PLoS One, 2013. **8**(7).

40. Li, Y., et al., *Clinical significance of EML4-ALK fusion gene and association with EGFR and KRAS gene mutations in 208 Chinese patients with non-small cell lung cancer.* PLoS One, 2013. **8**(1): p. 14.

41. Lee, H.Y., et al., *Favorable clinical outcomes of pemetrexed treatment in anaplastic lymphoma kinase positive non-small-cell lung cancer.* Lung Cancer, 2013. **79**(1): p. 40-5.

42. Gainor, J.F., et al., *ALK Rearrangements Are Mutually Exclusive with Mutations in EGFR or KRAS: An Analysis of 1,683 Patients with Non-Small Cell Lung Cancer.* Clin Cancer Res, 2013. **19**(15): p. 4273-81.

43. Desai, S.S., et al., *A year of anaplastic large cell kinase testing for lung carcinoma: Pathological and technical perspectives.* Indian J Cancer, 2013. **50**(2): p. 80-6.

44. Zhang, Y., et al., *Frequency of driver mutations in lung adenocarcinoma from female never-smokers varies with histologic subtypes and age at diagnosis.* Clin Cancer Res, 2012. **18**(7): p. 1947-53.

45. Yang, P., et al., *Worse disease-free survival in never-smokers with ALK+ lung adenocarcinoma.* J Thorac Oncol, 2012. **7**(1): p. 90-7.

46. Yoon, H.J., et al., *Repeat Biopsy for Mutational Analysis of Non-Small Cell Lung Cancers Resistant to Previous Chemotherapy: Adequacy and Complications.* Radiology, 2012. **28**: p. 28.

47. Wu, S.G., et al., *EML4-ALK translocation predicts better outcome in lung adenocarcinoma patients with wild-type EGFR.* J Thorac Oncol, 2012. **7**(1): p. 98-104.

48. Wang, Z., et al., *EML4-ALK rearrangement and its clinical significance in Chinese patients with advanced non-small cell lung cancer.* Oncology, 2012. **83**(5): p. 248-56.

49. Wallander, M.L., et al., *Comparison of reverse transcription-polymerase chain reaction, immunohistochemistry, and fluorescence in situ hybridization methodologies for detection of echinoderm microtubule-associated proteinlike 4-anaplastic lymphoma kinase fusion-positive non-small cell lung carcinoma: implications for optimal clinical testing.* Arch Pathol Lab Med, 2012. **136**(7): p. 796-803.

50. Takeda, M., et al., *Clinical outcome for EML4-ALK-positive patients with advanced non-small-cell lung cancer treated with first-line platinum-based chemotherapy.* Ann Oncol, 2012. **5**: p. 5.

51. Shaozhang, Z., et al., *Detection of EML4-ALK fusion genes in non-small cell lung cancer patients with clinical features associated with EGFR mutations.* Genes Chromosomes Cancer, 2012. **51**(10): p. 925-32.

52. Ren, S., et al., *Analysis of Driver Mutations in Female Non-Smoker Asian Patients with Pulmonary Adenocarcinoma.* Cell Biochem Biophys, 2012. **17**: p. 17.

53. Park, H.S., et al., *Immunohistochemical screening for anaplastic lymphoma kinase (ALK) rearrangement in advanced non-small cell lung cancer patients.* Lung Cancer, 2012. **77**(2): p. 288-92.

54. Kim, H.R., et al., *Distinct clinical features and outcomes in never-smokers with nonsmall cell lung cancer who harbor EGFR or KRAS mutations or ALK rearrangement.* Cancer, 2012. **118**(3): p. 729-39.

55. Just, P.A., et al., *Histologic subtypes, immunohistochemistry, FISH or molecular screening for the accurate diagnosis of ALK-rearrangement in lung cancer: a comprehensive study of Caucasian non-smokers.* Lung Cancer, 2012. **76**(3): p. 309-15.

56. Doebele, R.C., et al., *Oncogene status predicts patterns of metastatic spread in treatment-naive nonsmall cell lung cancer.* Cancer, 2012. **118**(18): p. 4502-11.

57. Li, C., et al., *Spectrum of oncogenic driver mutations in lung adenocarcinomas from East Asian never smokers.* PLoS One, 2011. **6**(11): p. 30.

58. Koh, Y., et al., *Clinicopathologic characteristics and outcomes of patients with anaplastic lymphoma kinase-positive advanced pulmonary adenocarcinoma: suggestion for an effective screening strategy for these tumors.* J Thorac Oncol, 2011. **6**(5): p. 905-12.

59. Shaw, A.T., et al., *Clinical features and outcome of patients with non-small-cell lung cancer who harbor EML4-ALK.* J Clin Oncol, 2009. **27**(26): p. 4247-53.

60. Han, X.H., et al., *Immunohistochemistry reliably detects ALK rearrangements in patients with advanced non-small-cell lung cancer.* Virchows Arch, 2013. **18**: p. 18.

61. Lee, J.O., et al., *Anaplastic lymphoma kinase translocation: a predictive biomarker of pemetrexed in patients with non-small cell lung cancer.* J Thorac Oncol, 2011. **6**(9): p. 1474-80.

62. Kobayashi, M., et al., *Detection of ALK fusion in lung cancer using fluorescence in situ hybridization.* Asian Cardiovasc Thorac Ann, 2012. **20**(4): p. 426-31.
