# Supplementary material for: Clinicopathological and Demographical Characteristics of Non-Small Cell Lung Cancer Patients with ALK Rearrangements: A Systematic Review and Meta-Analysis
Source: PLoS One. 2014 Jun 24;9(6):e100866. doi: 10.1371/journal.pone.0100866 (PMC4069179; doi:10.1371/journal.pone.0100866)
Supplement: Table S2 — ALK rearrangements and tumor stage. (DOC) [file pone.0100866.s013.doc]

**Table S2. ALK rearrangements and tumor stage**

| **Reference** | **Ethnicity** | **Stage (ALK^+^/Total)** | | | |
| --- | --- | --- | --- | --- | --- |
|  |  | **I** | **II** | **III** | **IV** |
| Yamaguchi N (2013) | Mixed | 1/18 | 0/12 | 2/29 | 20/193 |
| Zhang Y (2013) | Chinese | 5/166 | 1/104 | 5/105 | 9/98 |
| To K (2013) | Chinese | 8/154 | 4/46 | 3/86 | 7/87 |
| Xia N (2013) | Chinese | 3/41 | 3/20 | 5/46 | 0/3 |
| Li Ying (2013) | Chinese | 1/49 | 1/43 | 4/106 | 1/10 |
| Lee H (2013) | Korean | 1/40 | 3/39 | 6/133 | 10/162 |
| Zhou J (2013) | Chinese | 16/270 | 3/49 | 9/166 | 0/3 |
| Takeuchi K (2012) | Japanese | 21/905 | 8/195 | 14/339 | 1/37 |
| Han X (2013) | Chinese | NA | NA | 7/27 | 36/105 |
| Seo J (2012) | Korean | 5/138 | 0/27 | 2/27 | 1/6 |
| Paik J (2012) | Korean | 14/388 | 6/159 | 8/188 | NA |
| Fukui T (2012) | Japanese | 16/464 | 4/99 | 7/128 | 1/29 |
| Salido M (2011) | Spanish | 1/49 | 0/6 | 0/25 | 1/27 |
| Lee J (2011) | Korean | 3/9 | 0/2 | 1/13 | 11/71 |
| Zhang X (2010) | Chinese | 8/63 | 1/18 | 3/20 | 0/2 |
| Wong D (2009) | Chinese | 8/153 | 1/47 | 4/60 | 0/6 |
| Rodig S (2009) | Caucasian | 4/169 | 0/29 | 0/67 | 16/93 |
| Martelli M (2009) | European | 4/65 | 2/21 | 2/22 | 1/11 |
| Koivunen J (2008) | Mixed | 4/183 | 1/59 | 3/50 | 0/9 |
| Kobayashi M (2012) | Japanese | 3/380 | 1/74 | 4/111 | 0/16 |
| Zhang Y (2012) | Chinese | 6/206 | 3/33 | 5/99 | 1/11 |
| Yang P (2012) | Mixed | 14/175 | 4/16 | 10/62 | 6/47 |
| Wang Z (2012) | Chinese | NA | NA | NA | 11/113 |
| Doebele R (2012) | American | NA | NA | NA | 41/209 |
| Takeda M (2012­) | Japanese | NA | NA | 4/37 | 14/163 |
| Zhou S (2012) | Chinese | 4/34 | 0/17 | 3/40 | 1/11 |
| Ren S (2012) | Chinese | 5/51 | 1/14 | 3/24 | 1/15 |
| Kim H (2012) | Korean | 4/43 | 3/31 | 5/61 | 7/94 |
| Shaw A (2009) | Mixed | 2/25 | 0/1 | 0/9 | 17/96 |
| Jokoji R (2010) | Japanese | 4/179 | II-IV: 4/75 | | |
| Takamochi K (2013) (T) | Japanese | 1/116 | II-IV: 1/86 | | |
| Takamochi K (2013) (V) | Japanese | 2/114 | II-IV: 6/44 | | |
| Li Yuan (2013) | Chinese | 21/285 | II-IV: 23/287 | | |
| Inamura K (2009) | Japanese | 6/143 | II-IV: 5/110 | | |
| Paik P (2012) | Mixed | I-IIIA: 6/215 | | | IIIB/IV: 38/460 |
| Jin G (2012) | Korean | 4/93 | II-IIIA: 6/74 | | NA |
| Chen T (2012) | Chinese | I+II: 2/36 | | III+IV: 1/28 | |
| Wang R (2012) | Chinese | I+II: 19/388 | | III+IV: 17/245 | |

*Abbreviations*: NA, not available.
