# Supplementary material for: Clinicopathological and Demographical Characteristics of Non-Small Cell Lung Cancer Patients with ALK Rearrangements: A Systematic Review and Meta-Analysis
Source: PLoS One. 2014 Jun 24;9(6):e100866. doi: 10.1371/journal.pone.0100866 (PMC4069179; doi:10.1371/journal.pone.0100866)
Supplement: Table S3 — ALK rearrangements and NSCLC histology. (DOC) [file pone.0100866.s014.doc]

**Table S3. ALK rearrangements and NSCLC histology**

| **Reference** | **Ethnicity** | **Histology (ALK^+^/Total)** | | | | |
| --- | --- | --- | --- | --- | --- | --- |
|  |  | **Ad** | **SCC** | **ASC** | **LCC** | **Others** |
| Yamaguchi N 2013 | Mixed | 21/211 | 1/8 | NA | NA | 1/33 |
| Zhang Y 2013 | Chinese | 19/341 | 0/112 | 0/4 | 0/3 | 1/13 |
| Martinez P 2013 | Caucasian | 5/67 | 0/6 | NA | NA | 2/12 |
| Li Ying 2013 | Chinese | 6/95 | 0/96 | 1/7 | 0/0 | 0/10 |
| Lee H 2013 | Korean | 20/201 | 0/127 | NA | NA | 1/49 |
| Zhou J 2013 | Chinese | 25/349 | 2/101 | 1/18 | NA | 0/20 |
| Han X 2013 | Chinese | 42/128 | NA | 1/3 | 0/1 | NA |
| Dai Z 2012 | American | 38/1011 | NA | NA | NA | 11/376 |
| Conde E 2012 | European | 9/59 | 0/7 | NA | 0/4 | 0/16 |
| Soda M 2012 | Japanese | 32/524 | 0/141 | 0/5 | 0/9 | 0/75 |
| Paik J 2012 | Korean | 27/395 | 0/292 | NA | NA | 1/48 |
| Kobayashi M 2012 | Japanese | 8/381 | 0/143 | NA | NA | 0/57 |
| Kanaji N 2012 | Japanese | 4/88 | 0/62 | 0/1 | 0/1 | 1/9 |
| Jin G 2012 | Korean | 9/121 | 1/46 | NA | NA | NA |
| Chen T 2012 | Chinese | 2/36 | 0/23 | NA | NA | 1/5 |
| An S 2012 | Chinese | 10/130 | 4/93 | NA | 1/12 | 0/4 |
| Salido M 2011 | Spanish | 2/69 | 0/30 | NA | NA | 0/8 |
| Lee J 2011 | Korean | 14/78 | NA | NA | NA | 1/17 |
| Zhang X 2010 | Chinese | 10/62 | 2/29 | NA | 0/11 | 0/1 |
| Sakairi Y 2010 | Japanese | 7/82 | 0/18 | NA | NA | 0/9 |
| Wong D 2009 | Chinese | 11/209 | 0/34 | NA | NA | 2/23 |
| Martelli M 2009 | European | 3/63 | 4/48 | 2/2 | 0/3 | 0/4 |
| Boland J 2009 | American | 5/185 | 1/150 | NA | NA | NA |
| Shinmura K 2008 | Japanese | 2/50 | 0/20 | 0/3 | 0/4 | NA |
| Koivunen J 2008 | Mixed | 8/208 | 0/88 | 0/9 | NA | NA |
| Wang Z 2012 | Chinese | 10/95 | 1/18 | NA | NA | NA |
| Takeda M 2012­ | Japanese | 16/178 | NA | 1/2 | 1/6 | 0/14 |
| Zhou S 2012 | Chinese | 5/72 | 1/14 | 2/14 | NA | 0/2 |
| Kim H 2012 | Korean | 18/215 | 0/7 | NA | 1/3 | 0/4 |
| Doebele R 2012 | American | 38/200 | NA | NA | 1/5 | 2/6 |
| Shaw A 2009 | Mixed | 18/130 | 0/2 | 1/4 | NA | 0/5 |
| Sakai K 2012 | Japanese | 3/19 | NA | NA | 0/1 | NA |
| Takamochi K 2013 (T) | Japanese | 2/148 | 0/39 | 0/6 | 0/4 | 0/5 |

*Abbreviations*: Ad, adenocarcinoma; SCC, squamous cell carcinoma; ASC, adenosquamous carcinoma; LCC, large cell carcinoma; NA, not available.
