# Supplementary material for: Clinicopathological and Demographical Characteristics of Non-Small Cell Lung Cancer Patients with ALK Rearrangements: A Systematic Review and Meta-Analysis
Source: PLoS One. 2014 Jun 24;9(6):e100866. doi: 10.1371/journal.pone.0100866 (PMC4069179; doi:10.1371/journal.pone.0100866)
Supplement: Table S4 — The baseline characteristics of all qualified articles assessing both ALK rearrangements and EGFR/KRAS mutations. (DOC) [file pone.0100866.s015.doc]

**Table S4. The baseline characteristics of all included articles assessing both ALK rearrangements and EGFR/KRAS mutations**

| **References** | **Ethnicity** | **Histology** | **Mutation types** | **Mutated/Total** | **Coexist with ALK** | **Gender** | | **Age** | **Smoke-status** | | **Stage** | | | | **Histology** | | | |  |
| --- | --- | --- | --- | --- | --- | --- | --- | --- | --- | --- | --- | --- | --- | --- | --- | --- | --- | --- | --- |
|  |  |  |  |  |  | **Male** | **Female** |  | **Never smoker** | **Smoker** | **I** | **II** | **III** | **IV** | **Ad** | **Asc** | **Scc** | **Others** |  |
| Yamaguchi N (2013) | Mixed | NSCLC | ALK | 23/252 | NA | 12/98 | 11/154 | 56 (29-80) | 14/71 | 9/181 | 1/18 | 0/12 | 2/29 | 20/193 | 21/211 | NA | 1/8 | 2/41 |  |
|  |  |  | EGFR | 86/359 | 0 | 22/137 | 64/222 | 65 (33-90) | 45/102 | 41/257 | 3/23 | 6/22 | 7/45 | 70/260 | 84/310 | NA | 0/10 | 2/49 |  |
|  |  |  | KRAS | 71/207 | 2 | 22/75 | 49/132 | 65 (45-88) | 4/55 | 67/152 | 6/13 | 5/13 | 10/26 | 50/155 | 65/173 | NA | 0/9 | 6/34 |  |
| Gainor J (2013) | Mixed | NSCLC | ALK | 75/1683 | NA | 38 | 37 | 56 (29-87) | 49 | 26 | 4 | 8 | 18 | 45 | 71 | 0 | 3 | 1 |  |
|  |  |  | EGFR | 301/1683 | 0 | 86 | 215 | 64 (26-92) | 173 | 128 | 78 | 16 | 43 | 163 | 287 | 6 | 2 | 6 |  |
|  |  |  | KRAS | 465/1683 | 0 | 170 | 295 | 66 (26-92) | 18 | 445 | 175 | 55 | 69 | 165 | 432 | 9 | 4 | 20 |  |
| Li Y (2013) | Chinese | NSCLC | ALK | 7/208 | NA | 0/147 | 7 | 55 (42-71) | 6/78 | 1/130 | 1/49 | 1/43 | 4/106 | 1/10 | 6/95 | 1/7 | 0/96 | 0/10 |  |
|  |  |  | EGFR | 51/208 | 0 | 22/147 | 29/61 | 61.78 (33-83) | 32/78 | 19/130 | 10/49 | 9/43 | 26/106 | 6/10 | 42/95 | 3/7 | 5/96 | 1/10 |  |
|  |  |  | KRAS | 6/208 | 0 | 5/147 | 1/61 | 63.5 (53-72) | 1/78 | 5/130 | 0/49 | 2/43 | 3/106 | 1/10 | 4/95 | 0/7 | 1/96 | 1/10 |  |
| Li C (2011) | Chinese | Ad | ALK | 10/202 | NA | 4/43 | 6/159 | 59.3±9.8 | 10/202 | / | NA | NA | NA | NA | NA | NA | NA | NA |  |
|  |  |  | EGFR | 152/202 | NA | 30/43 | 122/159 | 58.3±9.8 | 152/202 | / | NA | NA | NA | NA | NA | NA | NA | NA |  |
|  |  |  | KRAS | 4/202 | NA | 3/43 | 1/159 | 62±3.4 | 4/202 | / | NA | NA | NA | NA | NA | NA | NA | NA |  |
| Xia N (2013) | Chinese | Ad | ALK | 11/110 | NA | 5/51 | 6/59 | NA | 11/81 | 0/29 | 3/41 | 3/20 | 5/46 | 0/3 | NA | NA | NA | NA |  |
|  |  |  | EGFR | 58/110 | 2 | 18/51 | 40/59 | NA | 54/81 | 4/29 | 22/41 | 12/20 | 23/46 | 1/3 | NA | NA | NA | NA |  |
|  |  |  | KRAS | 4/110 | 0 | 3/51 | 1/59 | NA | 1/81 | 3/29 | 1/41 | 0/20 | 3/46 | 0/3 | NA | NA | NA | NA |  |
| Zhou S (2012) | Chinese | NSCLC | ALK | 8/102 | NA | 2/54 | 6/48 | 51 (31-80) | 6/52 | 2/50 | 4/34 | 0/17 | 3/40 | 1/11 | 5/72 | 2/14 | 1/14 | 0/2 |  |
|  |  |  | EGFR | 44/102 | 0 | 19/54 | 25/48 | 62 (32-78) | 28/52 | 16/50 | 17/34 | 6/17 | 17/40 | 4/11 | 33/72 | 4/14 | 6/14 | 1/2 |  |
|  |  |  | KRAS | 17/102 | 1 | 10/54 | 7/48 | 59 (26-72) | 5/52 | 12/50 | 6/34 | 4/17 | 5/40 | 2/11 | 8/72 | 5/14 | 4/14 | 0/2 |  |
| An S (2012) | Chinese | NSCLC | ALK | 15/239 | NA | NA | NA | NA | 8/125 | 7/114 | NA | NA | NA | NA | 10/130 | NA | 4/93 | 1/12 |  |
|  |  |  | EGFR | 147/517 | 1 | NA | NA | NA | 119/291 | 28/226 | NA | NA | NA | NA | 140/347 | NA | 6/144 | 1/26 |  |
|  |  |  | KRAS | 27/498 | 0 | NA | NA | NA | 10/279 | 17/219 | NA | NA | NA | NA | 24/340 | NA | 2/132 | 1/26 |  |
| Doebele R (2012) | American | NSCLC | ALK | 41/209 | NA | 21/83 | 20/126 | 51 (21-78) | 31/84 | 10/125 | NA | NA | NA | 35/165 | 38/200 | NA | NA | 3/11 |  |
|  |  |  | EGFR | 39/209 | 0 | 10/83 | 29/126 | 62 (45-78) | 22/84 | 17/125 | NA | NA | NA | 28/165 | 37/200 | NA | NA | 2/11 |  |
|  |  |  | KRAS | 49/209 | 0 | 14/83 | 35/126 | 59.5 (32-82) | 6/84 | 43/125 | NA | NA | NA | 39/165 | 47/200 | NA | NA | 2/11 |  |
| KimH (2012) | Korean | NSCLC | ALK | 19 | NA | 2/30 | 17/199 | 59 (34-78) | NA | NA | 4/43 | 3/31 | 5/61 | 7/94 | 18/215 | NA | 0/7 | 1/7 |  |
|  |  |  | EGFR | 110 | 0 | 16/30 | 94/199 | 57 (33-78) | NA | NA | 24/43 | 19/31 | 25/61 | 42/94 | 105/215 | NA | 2/7 | 3/7 |  |
|  |  |  | KRAS | 8 | 0 | 0/30 | 8/199 | 61 (46-73) | NA | NA | 2/43 | 0/31 | 3/61 | 3/94 | 6/215 | NA | 0/7 | 2/7 |  |
| Koh Y (2011) | Korean | Ad | ALK | 45/221 | NA | 26 | 19 | 49 | 26 | 14 | NA | NA | NA | NA | NA | NA | NA | NA |  |
|  |  |  | EGFR | 46/135 | 0 | 23 | 23 | 63 | 29 | 17 | NA | NA | NA | NA | NA | NA | NA | NA |  |
| Kobayashi M (2012) | Japanese | NSCLC | ALK | 8/581 | NA | 2/375 | 6/206 | 56.6 | NA | NA | 3/380 | 1/74 | 4/111 | 0/16 | 8/381 | NA | 0/143 | 0/57 |  |
|  |  |  | EGFR | 191/581 | 0 | 66/375 | 125/206 | 63.8 | NA | NA | 141/380 | 18/74 | 25/111 | 7/16 | 185/381 | NA | 2/143 | 4/57 |  |
|  |  |  | KRAS | 56/581 | 0 | 38/375 | 18/206 | 62.9 | NA | NA | 34/380 | 5/74 | 12/111 | 5/16 | 45/381 | NA | 6/143 | 5/57 |  |
| Ren S (2012) | Chinese | Ad | ALK | 10/104 | NA | NA | NA | 54.0 (47-69) | NA | NA | 5/51 | 1/14 | 3/24 | 1/15 | NA | NA | NA | NA |  |
|  |  |  | EGFR | 73/104 | 0 | NA | NA | 60.0 (32-78) | NA | NA | 35/51 | 10/14 | 18/24 | 10/15 | NA | NA | NA | NA |  |
| Paik P (2012) | Mixed | Ad | ALK | 44/675 | NA | 22 | 22 | 60 (32-84) | 35/293 | 9/382 | I-IIIA | 6 | IIIB/IV | 38 | NA | NA | NA | NA |  |
|  |  |  | EGFR | 164/675 | 0 | 48 | 116 | 64.6 (39-88) | 110/293 | 54/382 | I-IIIA | 62 | IIIB/IV | 102 | NA | NA | NA | NA |  |
|  |  |  | KRAS | 171/675 | 0 | 50 | 121 | 65.4 (31-86) | 14/293 | 157/382 | I-IIIA | 54 | IIIB/IV | 117 | NA | NA | NA | NA |  |
| Lee J (2011) | Korean | NSCLC | ALK | 15/95 | NA | 7/44 | 8/51 | 52 (34-67) | 8/59 | 7/36 | 3/9 | 0/2 | 1/13 | 11/71 | 14/78 | NA | NA | 1/17 |  |
|  |  |  | EGFR | 43/95 | 0 | 20/44 | 23/51 | 63 (34-79) | 28/59 | 15/36 | 4/9 | 2/2 | 4/13 | 33/71 | 37/78 | NA | NA | 6/17 |  |
| Camidge D (2011) | American | NSCLC | ALK | 19/89 | NA | 9/31 | 10/58 | 46 | 16/43 | 3/46 | NA | NA | NA | NA | 18/83 | 0/2 | 0/1 | 1/3 |  |
|  |  |  | EGFR | 12/89 | 0 | 3/31 | 9/58 | 60 | 6/43 | 4/46 | NA | NA | NA | NA | 12/83 | 0/2 | 0/1 | 0/3 |  |
|  |  |  | KRAS | 21/89 | 0 | 6/31 | 15/58 | 58 | 3/43 | 18/46 | NA | NA | NA | NA | 19/83 | 0/2 | 0/1 | 2/3 |  |
| Martinez P (2013) | Caucasian | NSCLC | ALK | 7/85 | NA | 3/42 | 4/43 | 56.7 (38-78) | 5/30 | 2/55 | NA | NA | NA | NA | 5/67 | NA | 0/6 | 2/12 |  |
|  |  |  | EGFR | 13/99 | 0 | 3/51 | 10/48 | 63 (36-78) | 9/34 | 4/65 | NA | NA | NA | NA | 13/79 | NA | 0/7 | 0/13 |  |
| Seo J (2012) | Korean | Ad | ALK | 8/200 | NA | 2/91 | 6/109 | 56.3±11.6 | 8/116 | 0/80 | 5/138 | 0/27 | 2/27 | 1/6 | NA | NA | NA | NA |  |
|  |  |  | EGFR | 121/200 | 1 | 41/91 | 80/109 | 61.5±10.4 | 82/116 | 38/80 | 88/138 | 16/27 | 14/27 | 3/6 | NA | NA | NA | NA |  |
|  |  |  | KRAS | 24/200 | 0 | 18/91 | 6/109 | 67.0±8.7 | 10/116 | 11/80 | 16/138 | 3/27 | 4/27 | 0/6 | NA | NA | NA | NA |  |
| Zhang Y (2012) | Chinese | Ad | ALK | 15/349 | NA | NA | 15/349 | 54.5±8.8 | 15/349 | NA | 6/206 | 3/33 | 5/99 | 1/11 | NA | NA | NA | NA |  |
|  |  |  | EGFR | 266/349 | 0 | NA | 266/349 | 58.9±9.7 | 266/349 | NA | 166/206 | 24/33 | 68/99 | 8/11 | NA | NA | NA | NA |  |
|  |  |  | KRAS | 7/349 | 0 | NA | 7/349 | 51.9±15.1 | 7/349 | NA | 3/206 | 1/33 | 2/99 | 1/11 | NA | NA | NA | NA |  |
| Shaw A (2009) | Mixed | NSCLC | ALK | 19/141 | NA | 11/48 | 8/93 | 52 (29-76) | 14/59 | 5/82 | 2/25 | 0/1 | 0/9 | 17/96 | 16/89 | 0/2 | 1/4 | 2/46 |  |
|  |  |  | EGFR | 31/141 | 0 | 8/48 | 23/93 | 66 (36-90) | 21/59 | 10/82 | 10/82 | 0/1 | 2/9 | 26/96 | 24/89 | 0/2 | 0/4 | 7/46 |  |
| Wang R (2012) | Chinese | Ad | ALK | 36/633 | NA | 15/292 | 21/341 | NA | 26/408 | 10/225 | I+II: 19/388 | NA | III+IV: 17/245 | | NA | NA | NA | NA |  |
|  |  |  | EGFR | 387/633 | NA | 136/292 | 251/341 | NA | 296/408 | 91/225 | I+II: 253/388 | NA | III+IV: 134/245 | | NA | NA | NA | NA |  |
|  |  |  |  |  |  |  |  |  |  |  |  |  |  | |  |  |  |  |  |
| Takeda M (2012) | Japanese | NSCLC | ALK | 18/200 | NA | 9/127 | 9/73 | 46 (29-69) | 6/65 | 12/135 | IIIB 4/37 | | IV 14/163 | | 16/178 | 1/2 | NA | 1/20 |  |
|  |  |  | EGFR | 31/200 | 0 | 12/127 | 19/73 | 63 (44-75) | 16/65 | 15/135 | IIIB 5/37 | | IV 26/163 | | 30/178 | 0/2 | NA | 1/20 |  |

*Abbreviations:* Ad, adenocarcinoma; NSCLC, non-small-cell lung cancer; Age, mean ± SD or median (range); SCC, squamous cell carcinoma; ASC, adenosquamous carcinoma; LCC, large cell carcinoma; NA, not available.
